# Molecular Dynamics Simulation Framework to Probe the Binding Hypothesis of CYP3A4 Inhibitors

**DOI:** 10.3390/ijms20184468

**Published:** 2019-09-10

**Authors:** Yusra Sajid Kiani, Kara E. Ranaghan, Ishrat Jabeen, Adrian J. Mulholland

**Affiliations:** 1 Research Center for Modeling and Simulation (RCMS), National University of Sciences and Technology (NUST), Islamabad 44000, Pakistan; 2 Centre for Computational Chemistry, School of Chemistry, University of Bristol, Bristol BS8 1TS, UK

**Keywords:** docking, molecular dynamics simulation, CYP3A4, CYP3A4 inhibitors, WaterSwap, residue-wise energy decomposition

## Abstract

The Cytochrome P450 family of heme-containing proteins plays a major role in catalyzing phase I metabolic reactions, and the CYP3A4 subtype is responsible for the metabolism of many currently marketed drugs. Additionally, CYP3A4 has an inherent affinity for a broad spectrum of structurally diverse chemical entities, often leading to drug–drug interactions mediated by the inhibition or induction of the metabolic enzyme. The current study explores the binding of selected highly efficient CYP3A4 inhibitors by docking and molecular dynamics (MD) simulation protocols and their binding free energy calculated using the WaterSwap method. The results indicate the importance of binding pocket residues including Phe57, Arg105, Arg106, Ser119, Arg212, Phe213, Thr309, Ser312, Ala370, Arg372, Glu374, Gly481 and Leu483 for interaction with CYP3A4 inhibitors. The residue-wise decomposition of the binding free energy from the WaterSwap method revealed the importance of binding site residues Arg106 and Arg372 in the stabilization of all the selected CYP3A4-inhibitor complexes. The WaterSwap binding energies were further complemented with the MM(GB/PB)SA results and it was observed that the binding energies calculated by both methods do not differ significantly. Overall, our results could guide towards the use of multiple computational approaches to achieve a better understanding of CYP3A4 inhibition, subsequently leading to the design of highly specific and efficient new chemical entities with suitable ADMETox properties and reduced side effects.

## 1. Introduction

Drug metabolism has become a focus for research due to its important role in understanding pharmacotoxicology, pharmacotherapy and applications in drug delivery [1,2,3,4]. The human Cytochrome P450 family of heme-containing enzymes are one of the most important classes of enzymes involved in drug metabolism due to their major role in phase I metabolic reactions (N- hydroxylation, sulphoxidation, epoxidation, deamination, desulfuration, dehalogenation, peroxidation, O- and S-dealkylation) of various endogenous and exogenous compounds including drugs [5,6]. The CYP3A4 subtype from the CYP3A family is one of the most dominant drug metabolizing enzymes in humans, responsible for the metabolism of ~50% of currently marketed drugs [7]. The CYP isoforms show an inherent ability for the metabolism of structurally diverse molecules which ultimately leads to the poor bioavailability of various drugs [8,9]. Moreover, CYP activity in intestinal and liver microsomes has been associated with a high number of documented drug–drug and drug–food interactions [10,11,12]. The combination of high abundance in the liver and a large active site cavity capable of the simultaneous binding of multiple diverse substrates [13,14] means that it is implicated in undesirable drug–drug interactions and toxicological outcomes [15]. Drug–drug interactions arise mainly due to the inhibition or induction of human CYPs where inhibition might reduce the metabolism and elimination of drugs leading to toxicological outcomes, and induction might compromise the bioavailability and therapeutic efficiency [16]. Similarly, the induction of CYP3A4 might lead to reduced bioavailability due to rapid degradation whereas the inhibition of CYP3A4 leads to increased drug levels and toxicity [17]. Therefore, it is of great importance to understand the underlying mechanisms behind the induction and inhibition of major metabolic enzymes including CYP3A4 so that the impact of these processes can be minimized.

Generally, to probe the molecular basis of CYP450 related metabolism, inhibition and drug–drug interaction potential a number of ligand-, structure-based and integrated modeling studies have been reported in literature [18,19,20,21,22,23,24,25]. Particularly, to gain deeper insights into the binding of ligands to CYP3A4, various in silico studies based on quantitative structure–activity relationships (QSAR), pharmacophore models and machine learning methods have been undertaken [26,27,28,29,30,31]. Structure-based approaches also provide efficient computational methods for the investigation of the interactions responsible for ligand binding to a particular target which is indispensable for understanding metabolic processes and the development of novel leads and therapeutic agents [32]. The availability of X-ray crystallographic structures has also provided a sound basis for computational studies exploring the catalytic mechanism and estimating the binding properties of small molecules with mammalian CYP3A4 [17]. Docking, molecular dynamics simulations and binding energy predictions for the inhibitors and substrates of CYP3A4 have been described extensively [18,33,34,35,36]. The binding affinity metric is important and advantageous over expensive experiments for the assessment of ligand interactions with the binding site residues and the desolvation effects [37,38]. For a ligand–protein complex, various methods are available for the prediction of binding energy [39]. In the current study, a combination of docking, molecular dynamics simulations (MD) and binding free energy predictions (using the WaterSwap method [40]), molecular mechanics/Poisson–Boltzmann surface area (MM/PBSA) and molecular mechanics/generalized-Boltzmann surface area (MM/GBSA) [41,42] have been performed for a set of highly efficient CYP3A4 inhibitors to investigate CYP3A4 inhibition. The ligand affinity for CYP3A4 and the overall contribution of the binding site residues towards the stabilization of the inhibitor-bound complex can be identified through WaterSwap calculations, insight which might aid the development of new therapeutic options by reducing drug–drug interactions and the associated undesirable toxic effects.

## 2. Results and Discussion

### 2.1. Protein Structure Selection

**Figure 1:**
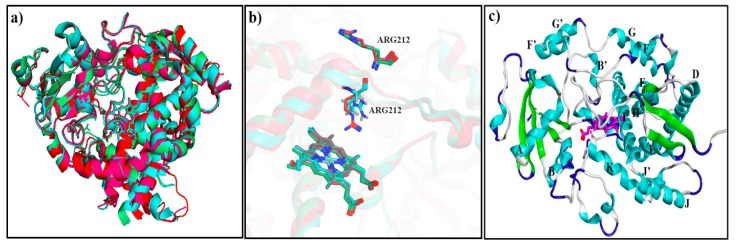
Superposition of chain A in CYP3A4 crystal structures (**a**) 1TQN (red), 3UA1 (cyan), 3NXU (green) and 4K9W (pink); (**b**) positioning of Arg212 within 1TQN (red), 3UA1 (cyan), 3NXU (green) and 4K9W (pink); and (**c**) the structural organization of 1TQN.

To date, about 25 CYP3A4 crystallographic structures in ligand-bound and ligand-free forms are available in the Protein databank and the structures 3NXU (2 Å) [43], 1TQN (2.05 Å) [44], 3UA1 (2.15 Å) [45] and 4K9W (2.40 Å) [46] were selected for further conformational analysis. 1TQN is a structure of the apo-state of the enzyme, whereas the remaining three structures represent ligand-bound states. Moreover, a few N- and C-terminal residues were also missing in all structures. In addition, 3NXU has missing G-H loop residues (Glu264-His267) and H-I loop residues (Ser281-Lys288), 1TQN has missing H-I loop residues (Lys282-Glu285), whereas 3UA1 has missing G-H loop residues (Ser259-Leu261, Lys266-Arg268) and H-I loop residues (Asn280-Ser286) and 4K9W has a missing region of G-H loop (Gln265-His267) and H-I loop residues (Ser281-His287). The structure 1TQN, with fewer missing residues (only at H-I loop in addition to the missing -N/-C terminal residues) was selected as a template for the calculation of root-mean-square deviation of the C_α_ atom’s (C_α_RMSD) in comparison to the 3NXU, 3UA1 and 4K9W structures. Overall, a C_α_RMSD of 1.28 Å of 1TQN as compared to 3NXU, 3UA1 and 4K9W shows that the backbone conformations of these structures are similar (Figure 1a). It is notable that in 1TQN and 3UA1, Arg212 is positioned in the vicinity of heme within the active site, whereas in the other ligand-bound structures it is pointing outwards (Figure 1b). The positioning of Arg212 towards the active site is implicated to be important in terms of substrate/inhibitor binding within the CYP3A4 binding site [45,47]. Therefore, due to a reasonable RMSD of 1TQN to 3UA1 (0.52 Å) and all other structures, fewer missing residues and the position of Arg212 towards binding site, 1TQN was selected as the final structure after building the missing residues using Modeller [48]. The overall structural organization of 1TQN is shown in Figure 1c and it is notable that the helices F-F’ and G-G’ form the roof of the heme embedded binding site.

### 2.2. Ligand Selection

**Figure 2:**
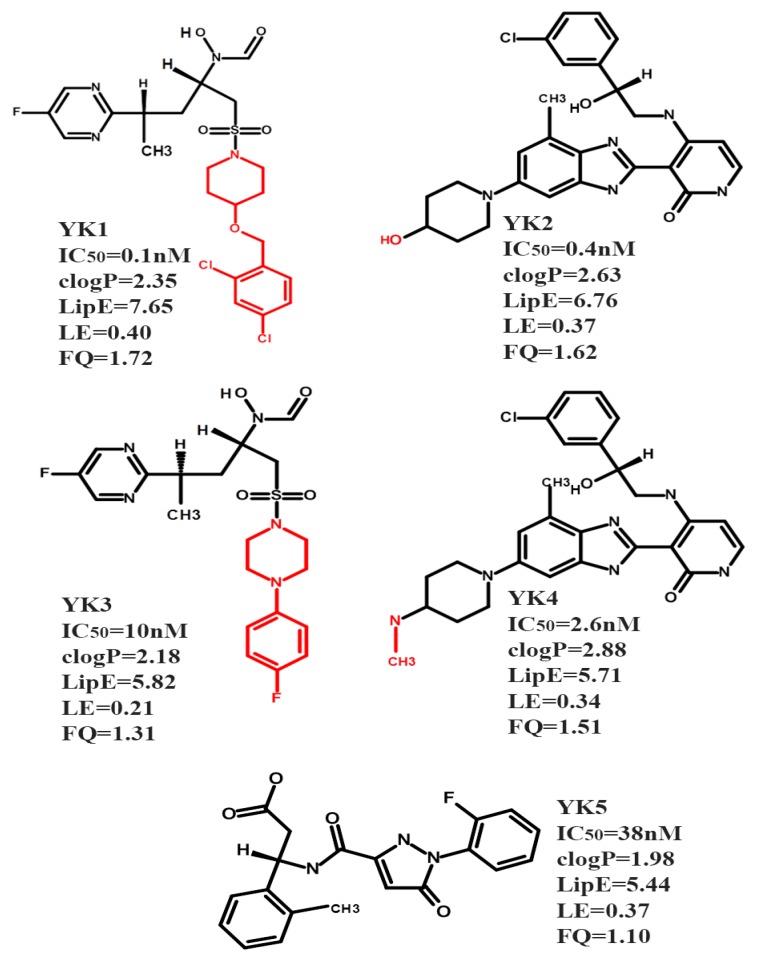
The chemical structures and experimental biological activity (IC_50_) values of the selected CYP3A4 inhibitors fulfilling the drug efficiency criteria. For structural analogues the common scaffold is shown in black and the R groups are presented in red. The selected CYP3A4 inhibitors YK1–5 have the following ChEMBL IDs CHEMBL1683444 (YK1), CHEMBL520419 (YK2), CHEMBL1683445 (YK3), CHEMBL482102 (YK4) and CHEMBL3145341 (YK5) and are highlighted yellow in Table S1.

Initially, the concept of drug efficiency metrics has been applied for the selection of efficient modulators against true drug targets [49]. Recently, drug efficiency metrics were used to select the highly efficient inhibitors of CYP450 isoforms which represents an anti-target [22]. Only five CYP3A4 inhibitors (Figure 2) out of the large data set of 2747 compounds (Table S1) showing the defined thresholds (Materials and Methods and Section S1) of activity (IC_50_: 0.1–38 nM), clogp (1.98–2.88), lipophilic efficiency (LipE) (5.44–7.65), ligand efficiency (LE) (0.21–0.40 kcal mol^−1^ HA-1) and fit quality ≥1 (Figure S1a,b) were selected for further docking and molecular dynamics studies to probe the binding hypothesis within the active site of CYP3A4.

### 2.3. Docking and Molecular Dynamics Simulation (MD)

Our aim here is not to investigate drug–drug interactions, but rather to investigate the potential binding modes of the chosen CYP3A4 inhibitors: this is the initial issue and primary question of interest in drug development process, i.e., is it likely that a given compound will inhibit CYP3A4. A secondary question, which is relevant for compounds to be used in the clinic, is whether drug–drug interactions may be expected, and how they occur as reported for warfarin by Lonsdale et al. [35]. This might be an interesting question for similar studies in the future. Therefore, the primary aim of our study is the elucidation of the binding hypothesis of the selected inhibitors using the experimentally determined structure of the soluble portion of the CYP3A4 enzyme (1TQN). Generally, the CYP450 microsomal enzymes are partly immersed in the membrane and anchored through the N-terminal helix. The heme shows a tilt angle due to its orientation with reference to the plane of membrane and the F’/G’ loop is deeply buried while the B/C-loop and β1, β2, β4 andβ5 sheets also show substantial contacts s with the membrane [50,51]. The CYP proximal site shows an interface with the cytosol while the distal site where the substrate ingress and egress channels are located is positioned towards the lipid bilayer [52]. Docking, molecular dynamics and QM/MM approaches have remained fruitful for the elucidation of oxidation selectivity of substrates whereas to bring about complete perspective of drug metabolism other key factors, such as substrate access through the membrane and binding to the active site, must be taken into consideration [53,54].

Previously, several authors have investigated the effects of including the membrane on binding with major CYP subtypes and it was shown that in comparison to crystallographic structures and single solution simulations the consideration of membrane binding instigates conformational changes to the substrate ingress and egress channels at the distal site [35,51,52,55,56]. We have also previously investigated the effects of including the membrane (and the membrane binding domain) on binding in CYP3A4 [35]. Comparisons of simulations with and without the membrane present showed that the inhibitor binding mode is not significantly affected by the presence of the membrane; as there was a minimal effect on the protein conformational flexibility and residues in the near vicinity of heme proximal site yet, significant differences were observed in the substrate access channels of both systems [35]. However, for other properties (e.g., pathways of association and binding of inhibitors or substrates that pass through the membrane), in contrast, consideration of the membrane is essential [35]. It is also well known, that N-terminal α-helix connects the human CYPs to the membrane, but due to difficulties in dealing with CYP-membrane-bound structures and to ease the process of crystallization, hydrophilic residues are added as a replacement of this helix [17]. It is also observed that the deletions in the crystallized structures did not reduce the catalytic effect or the ability of the enzyme to attach to membrane surface hence, these engineered structures are biochemically relevant [57,58]. Therefore, owing to the complexity of dealing with CYP-membrane-bound structures, this approach of modeling the soluble domain only has been applied successfully in investigations of inhibition and activity of CYP3A4 and other P450s [36,59,60,61,62].

Herein, the docking and molecular dynamics simulations are based on the experimentally determined structure of the soluble part of the enzyme. The AutoDock [63] molecular docking platform was used to generate a maximum of 10 different binding conformations per ligand. The concept of alternative binding modes is clearly very important for binding in particular with the highly plastic, CYP3A4 active site [47]. However, for our dataset, a careful inspection of the observed docking conformations of the selected CYP3A4 inhibitors showed no evidence of binding to multiple sites within the CYP3A4 active site rather, the docked conformations depict clustering at a similar position with reasonable RMSD values (1.46–3.5 Å) as shown in Figure S2a–e. Therefore, as reported in various studies the minimum energy pose ranked through the free energy scoring function was selected for further analysis [36,64]. The docking results of the selected ligand–protein complexes including Autodock energy (kcal/mol), the residues involved in van der Waals interactions and hydrogen bonding in the docked structures are given in Table 1.

AMBER14 [65] was used to explore the conformational flexibility and stability of the inhibitor-bound complexes as described in the Materials and Methods section. The trajectory analysis was performed using CPPTRAJ [65] through the determination of root-mean-square deviation of the C_α_ atoms (C_α_RMSD), root-mean-square fluctuation (C_α_RMSF), radius of gyration (Rg) and hydrogen bond analysis for each complex. For the selected five CYP3A4 inhibitor-bound complexes multiple runs of MD (two for each complex) were performed. The conformational analysis of the replicated MDs indicates that the MD results do not differ significantly as shown by difference of ~0.09 to 0.57 Å and ~0.01 to 0.07 Å in the C_α_RMSD and the Rg values for the ligand-bound complexes (CYP3A4-YK1–5) (for detailed analysis refer to Supplementary Materials Section S2 and Figure S3). Therefore, the initial run of MDs (MD1) with smaller average C_α_RMSD and Rg values were considered final for further analysis. The RMSD of C_α_ atoms in the apo-state of CYP3A4 and the five selected ligand-bound models was measured as a function of time with respect to the initial structure (Figure S4). For the unliganded CYP3A4 (1TQN) protein, high fluctuations were observed in the C_α_RMSD up to 10 ns, after which it stabilized at an average C_α_RMSD value of 2.83 (±0.4) Å. However, the ligand-bound simulations took longer to stabilize, with high fluctuations in the RMSD over the first ~25–30 ns for CYP3A4-YK1 and CYP3A4-YK4, and ~35 ns for the CYP3A4-YK2, CYP3A4-YK3 and CYP3A4-YK5 complexes (Figure S4). Overall, with the exception of the CYP3A4-YK1 (average C_α_RMSD: 3.13 (±0.7) Å) the remaining complexes CYP3A4-YK2-YK5 displayed average C_α_RMSD values of 2.23 (±0.4) Å, 2.11 (±0.6) Å, 2.09 (±0.3) Å and 2.31 (±0.7) Å, respectively, that lie in a comparable range to the average C_α_RMSD (2.83 (±0.4) Å) of the apo-protein (1TQN) indicating the stability of all the selected systems.

The C_α_ based RMSF values of the ligand-bound complexes show the highly plastic or stable regions of the proteins during the course of MD simulations. For all five inhibitor-bound complexes our results indicate a fluctuation pattern similar to that obtained for the apo-protein, showing the highest fluctuations in flexible regions including the channel, loop forming regions, the N and C terminal regions as shown in Figure S5. Overall, for all systems a few core regions and the loop regions including the G-H and the built-in missing H-I loop demonstrated the highest RMSF values (within 2.0–5.4 Å). After the assessment of conformational changes in the CYP3A4 structure upon ligand binding through RMSF calculations, the radius of gyration was also measured for the apo-protein and the selected systems to predict the overall compactness of the CYP3A4-inhibitor-bound complexes. The radius of gyration (Rg) parameter shows the equilibrium conformation of a total system and is calculated as the root-mean-square distance of an assemblage of atoms from their common center of mass [66]. The Rg of the apo-protein 1TQN shows a highly compact protein with an average Rg value of 44.3 (±0.03) Å for the 50 ns MD, with the ligand-bound complexes giving very similar values at 44.3 (±0.05) Å, 44.4 (±0.06) Å, 44.5 (±0.1) Å, 44.3 (±0.03) Å and 44.4 (±0.04) Å for complexes of YK1–5, respectively. Overall, the Rg values of the complexes CYP3A4-YK1–5 show that upon ligand binding the CYP3A4 retains a compact conformation as shown in Figure S6.

Detailed interaction profiles of the selected CYP3A4 inhibitors with important binding site residues of CYP3A4 before and after 50 ns of MD, along with the centroid structures for each complex, are provided in Table 1. Here, the centroid structures for all CYP3A4-inhibitor bound complexes were obtained after performing clustering on the basis of RMSD values of important active site residues as described in the Material and Methods section. It is notable that for the selected inhibitors, the docking energies vary from −11.7 to −9.5 kcal/mol, a fairly narrow range for a series of ligands that span one order of magnitude in terms of IC_50_ values and the predicted ordering of the ligands shows little correlation with the experimental data (Figure S10a). Phe57, Arg105, Ser119, Arg212, Phe215, Thr309, Ser312, Ala370, Arg372 Glu374 and Leu483 were observed to form significant interactions with the ligands (Table 1). The selected docking poses of the inhibitors (YK1 to YK5) were positioned in the close vicinity of the buried helix I, near the heme and below the roof formed by the loop region between the F’- F and G’- G helices. The docking and MD results of the selected compounds (YK1–5) show no evidence of direct coordination to iron (i.e., type II binding), indicating that such close approach does not occur for any of these compounds. However, the known limitations of docking methods, and of empirical (MM) force fields for modeling metalloprotein interactions, are well known and should be considered. Modeling such interactions where the inhibitor may bond directly to iron, would require sophisticated methods capable of representing a bonding interaction, e.g., QM/MM calculations employing good quality density functional theory [35,53,67,68,69]. Whereas, the approaches used here allowing for movement of the ligand indicate that this mode of binding is not likely.

Overall, a close assessment of the binding modes of inhibitors (YK1–5) show that YK1, YK2, YK3 and YK4 protrude from the proximal binding site towards the distal site (Figure S8a,c,e,i) whereas, YK5 binds at the distal binding site (Figure S8g) with the phenylalanine-cluster (Phe108, Phe215, Phe219, and Phe304), Ser119 and arginine residues (Arg105, Arg106, Arg212, Arg372) contributing significantly towards interactions (Table 1). The docked ligands YK1–5 within the proximal and distal sites of CYP3A4 active site are shown in Supplementary Materials Figure S7a–e as discussed by Tanaka et al. [70]. Residues including Ser119, Ile301, Phe304, Ala305, Ile369, Ala370 and Glu374 have been identified as important for binding through site-directed mutagenesis and X-ray crystallographic studies [44,71,72,73,74]. Likewise, our docking results are well in line with the previously reported studies that have shown the importance of active site residues Val101, Asn104, Arg105, Met114, Ser119, Leu211, Arg212, Asp214, Asp217, Pro218, Glu374, Ile301, Ala305, Thr309, Ile369, Ala370, Leu373, Glu374, Ser478, Leu479 and the Phe-cluster for interaction with the inhibitors and substrates of CYP3A4 [33,44,71,74,75]. MD simulations show that the hydrogen bonds identified in the docking poses did not persist throughout the trajectory; however, new hydrogen bonds are formed during the course of the MD simulations. The time dependent hydrogen bond analysis of CYP3A4-inhibitor complexes during the 50 ns simulation demonstrates that the highly potent inhibitors YK1 and YK2 form the highest numbers of hydrogen bonds in comparison to inhibitors YK3-YK5 as shown in Figure S9a–e.

**Figure 3:**
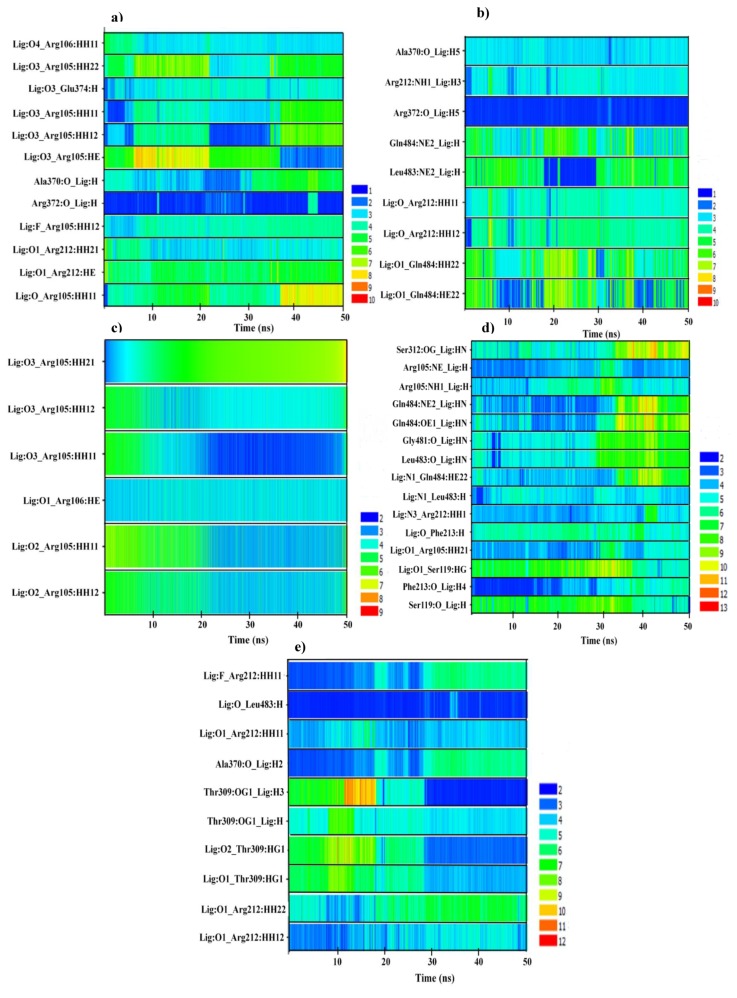
The time dependent distance analysis of (**a**) CYP3A4-YK1; (**b**) CYP3A4-YK2; (**c**) CYP3A4-YK3; (**d**) CYP3A4-YK4; and (**e**) CYP3A4-YK5 residues and inhibitor groups involved in hydrogen bonding. The distances are measured in Angstrom (Å) and the time is shown along the x-axis.

The analysis of hydrogen bond distances between CYP3A4 and YK1 shows that the hydrogen bonds with Arg105and Arg212 in the docked complexes did not persist during the MD. However, in the CYP3A4-YK1 centroid structure, hydrogen bonds were identified between YK1:Carbonyl=O3-Glu374:NH and YK1:Hydroxyl-O2-Arg372: =O (Figure S8b) which remained stable till the end of the 50 ns MD simulation (Table 1). Other residues including Arg105, Arg106, Arg212 and Ala370 formed hydrogen bonds only during part of the MD simulation as shown in Figure 3a. For YK3, a structural analogue of YK1, three hydrogen bonds were observed between the inhibitor and Arg105, Arg106 and Arg372 of the centroid structure (Figure S8f). Figure 3c represents the time dependent distance analysis of the hydrogen bonds formed between YK3 and Arg105–106 during the 50 ns MD simulation. The hydrogen bond between YK3:O3-Arg105:HH1 remained stable during the last 30 ns of the MD simulation (Figure 3c).

Additionally, the CYP3A4 inhibitors YK2 and YK4, yet another pair of structural analogues, formed hydrogen bonds with Ser312 in the pose from docking (Figure S8c,g), whereas in the centroid structures, YK2 and YK4 formed one and three hydrogen bonds, respectively (Table 1, Figure S8d,h). However, after MD, hydrogen bonds between YK2:Hydroxyl-O2- Arg372:O and YK4: Hydroxyl-O1-Ser119:O were observed as shown in Table 1. The time series distance profiles of CYP3A4-YK2 and CYP3A4-YK4 during the 50 ns MD simulations show that the hydrogen bonds formed between Arg372:O-YK2:H5 remained stable throughout the trajectory and the bond between Phe213:O-YK4:H4 was observed for a short duration as shown in Figure 3b,d. Prior to MD, YK5 displayed hydrogen bonding interactions with Arg212 and Glu374 (Table 1 and Figure S8i), whereas, in the centroid structure Arg212, Ala370 and Leu483 were involved in hydrogen bonding (Table 1 and Figure S8j). A stable hydrogen bond between Leu483:H-YK5:O was observed in the MD trajectory and hydrogen bonds between Arg212:HH11-YK5:F and Thr309:OG1-YK5:H3 were only stable for a fraction of the simulation. The distance of the hydrogen bonds between amino acid residues and YK5 functional groups are shown in Figure 3e.

Generally, the MM(GB/PB)SA method is used for the calculation of binding free energy by selecting snapshots at regular intervals over an entire MD simulation and it does not take into account the protein–water and ligand–water interaction details as it uses an implicit water model. The WaterSwap method uses explicit water molecules [76], so does not suffer from the same limitations as the MM(GB/PB)SA method. The WaterSwap method has been applied successfully to other systems [77,78,79,80,81,82,83,84]. Herein, we apply the WaterSwap method to estimate the binding free energies for the five highly potent inhibitors of CYP3A4. Overall, the WaterSwap calculations for the five selected complexes gave binding energies in the range −46.7 (±0.5) to −30.9 (±0.6) kcal/mol as shown in Table 2, indicating that the inhibitors all bind strongly to CYP3A4. The free energy values calculated through the BAR, FEP and TI methods differ by ≤ 1 kcal/mol indicating good convergence of the calculations. Two values are reported for YK4 and YK5 as the cluster analysis showed that structures belonging to the dominant cluster did not exist across the entire trajectory, so the centroid of the second dominant cluster was also included in the analysis. The binding energies predicted for YK4 are −39.7 (±0.5) kcal/mol and −30.9 (±0.6) kcal/mol for the centroid structures of the first two dominant clusters from the MD simulations and for YK5 the corresponding values are −36.0 (±0.4) kcal/mol and −40 (±0.3) kcal/mol, respectively. For YK4, a decrease of ~8.8 kcal/mol was observed in the calculated binding energy for the second centroid structure because the residues (Arg106, Arg212, Arg372 and Gln434) previously favoring the stabilization of the inhibitor–protein complex of the first centroid structure now contribute significantly towards the stabilization of the water cluster as shown in Figure 5. However, for YK5 a higher binding energy for the second centroid structure might be due the stabilization of the inhibitor–protein complex by residues including Arg106, Arg212, Glu308, Thr309 and Gly481 that were contributing negatively towards inhibitor–protein stabilization in the previous calculation (Figure 5). There is a significant difference in energy for the two starting geometries, showing that even though there will be some configurational sampling through the Monte Carlo simulations, care should be taken in the selection of a starting geometry for the WaterSwap calculations.

Furthermore, binding energy calculations were performed using the Molecular Mechanics/Generalized Born Surface Area (MM/GBSA) and Molecular Mechanics/Poisson–Boltzmann (MM/PBSA) methods, to analyze the interactions of the selected CYP3A4 inhibitors with the binding site residues (by taking into account both entropic and enthalpic contributions) and for the comparative analysis of the binding energies calculated through the WaterSwap method [41,42]. The binding energy calculations through these methods are reproducible and computationally inexpensive with a relatively good accuracy [85]. The calculated binding free energies extracted from 100 snapshots of the MD trajectories were averaged. The binding energy values calculated by the MM/PBSA and MM/GBSA methods were predicted in the range −40.78 to −22.52 kcal/mol and −61.22 to −34.96 kcal/mol, respectively, as shown in Table 2. The important energy components of these calculations are shown in Supplementary Materials Table S2. Although the difference in solvation and gas phase Gibbs free energy is similar between the two methods but the comparison of binding energy predictions for the selected CYP3A4-inhibitor complexes indicates that higher binding energies were obtained by MM/GBSA method which might be due to the fact that MM/PBSA has an additional dispersion free energy component for calculation of binding energy and due to the difference in ΔE_EGB/EPB_ and ΔG_solv_ values in both methods. Overall, for all selected complexes the MM/PBSA and MM/GBSA calculations show that the van der Waals and electrostatic interactions had the highest contribution towards the binding energy as shown in Table S2.

**Figure 4:**
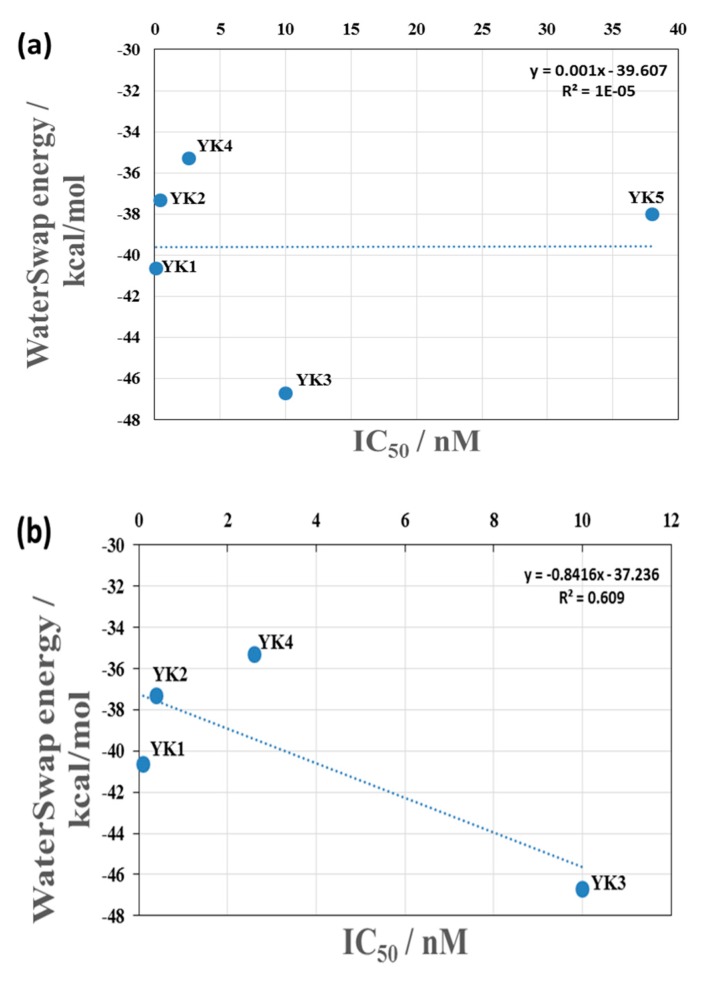
(**a**) The correlation between the binding free energy of the complex predicted by WaterSwap and the IC_50_ values for YK1–5. (**b**) The correlation between the binding free energy of the complex predicted by WaterSwap and the IC_50_ values with the data for YK5 removed as it is a significant outlier compared to the other compounds. Note that the values for YK4 and YK5 are the average of WaterSwap calculations using two different starting structures to ensure that the structures were representative of the entire MD trajectory.

For the selected CYP3A4-inhibitor complexes docking scores show that, this is a relatively small range of energies compared to the order of magnitude difference in the IC_50_ values of the inhibitors. Figure 4a shows a plot of the binding free energy (calculated through WaterSwap) vs IC_50_ value for the complexes, where it can be seen that there is no correlation with the experimental data. Figure 4b shows the same plot but with the data for YK5 removed as, with an IC_50_ value of 38 nM, it lies far outside the values of the other inhibitors. The correlation between the binding free energy and experimental IC_50_ value is significantly improved with a correlation coefficient of R^2^ = 0.6 (when an average of the two values are used for YK4). Correlation of the docking scores with the experimental data is also improved by the omission of the YK5 data, but the correlation remains weak (R^2^ = 0.31) as shown in Figure S10b. Overall, comparison of the binding energies obtained using MM/GBSA, MM/PBSA and WaterSwap methods shows that the calculated values do not differ significantly. However, MM/GBSA and MM/PBSA based binding energy values show little or no correlation with the IC_50_ values of the selected compounds (as shown in Figure S11a,b). The removal of compound YK5, a significant outlier in terms of activity, did not improve the R^2^ values for both methods. Therefore, WaterSwap provides a good estimate of the general trends for these compounds and is better than using the docking score alone; however, it is not very good at accounting for the differences in binding for structurally quite similar compounds. YK1 is a better inhibitor than YK3 by an order of magnitude in terms of IC_50_ value, but YK3 has a higher calculated binding free energy than YK1. A larger test set of ligands is needed to fully benchmark the ranking of ligands in terms of binding free energy.

**Figure 5:**
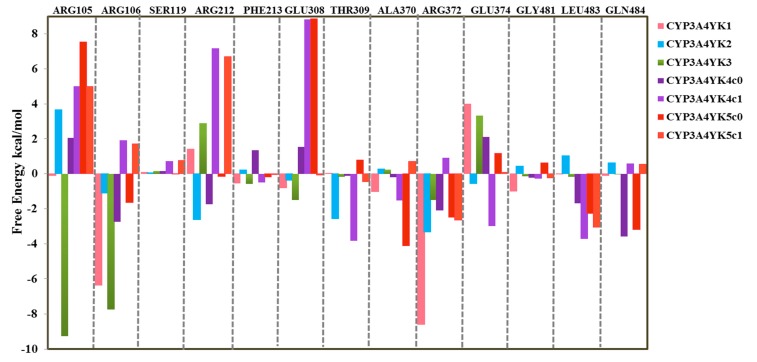
The decomposition of the WaterSwap binding free energy into residue-wise components. The values for the selected CYP3A4 inhibitors are shown, where negative values indicate that the residue stabilizes the inhibitor–protein complex and positive values indicates stabilization of the water cluster rather than the inhibitor.

Raza et al. have shown that in comparison to the MM/PBSA analysis, the WaterSwap method identifies more residues with significant contribution to the calculated binding energies [86]. Additional insight can be obtained from the WaterSwap calculations as the binding free energy can be decomposed in terms of individual residue contributions. It is important to emphasize that the interaction energies calculated through this method are not directly comparable to experimental results (e.g., ΔΔ*G* for mutated enzymes) nor will they sum to the total binding energy; however, this type of decomposition analysis might be useful for the identification of residues that have the largest effects on the binding energy. The residue decomposition can be visualized and analyzed using the CHEWD plugin for Chimera or PyMol [86]. The residue decomposition indicates favorable and unfavorable interactions, information that is useful for lead development. Here, the WaterSwap residue-wise binding energy decompositions were analyzed for the residues with greatest contributions to the inhibitor binding throughout the course of MD simulations. These include 13 residues (Arg105, Arg106, Ser119, Arg212, Phe213, Glu308, Thr309, Ala370, Arg372, Glu374, Gly481, Leu483, and Gln484) for which the total G_residue_ components are shown in Figure 5. For YK1–5 the binding energy calculations based on the first centroid clusters show negative free energies of Arg106 and Arg372 for all five selected CYP3A4 inhibitors, favoring the stabilization of the inhibitor–protein complexes through van der Waals and hydrogen bond interactions. Whereas, the binding energy calculations for YK4 based on the second cluster show positive free energies for both Arg106 and Arg372 and only Arg106 for YK5 (Figure 5).

YK1 has strong interactions with both arginines 106 and 372, whereas YK3 has a strong interaction with Arg106 and a weaker interaction with Arg372 but has an additional very favorable interaction with Arg105 not seen in the other complexes. This additional stabilization from Arg105 may be the reason why YK3 has a greater binding free energy than YK1. The residue contribution for Ser119 is very small in all complexes, weakly positive for complexes of CYP3A4 with YK1–4, showing a slight preference for the water rather than the ligand, and weakly negative for YK5 (first cluster c0). The interaction between the ligand and Glu374 is unfavorable in all complexes except CYP3A4-YK2 and CYP3A4-YK4 (second cluster c1), where it is slightly favorable due its involvement in van der Waals interactions. For both structurally similar inhibitors YK2 and YK4, the binding energy calculations based on the first cluster show favorable interactions with Arg212 which are not present in complexes of YK1 and YK3. For YK4 the calculated values using both clusters also show stabilizing interactions with Leu483 whereas, Gln484 favors the stabilization of the inhibitor–protein complex for the first cluster only. YK5 has the smallest binding free energy of all the ligands studied when using the first cluster for calculation, and the residue-wise decomposition shows that it does not have as strong favorable interactions with the arginine residues in the binding site and that the most significant residues for binding in this complex are Ala370 and Gln484. Overall, Arg106 and Arg372 provide the most significant contribution to the binding of the inhibitors studied here.

The availability of CYP crystallographic structures and the use of various in silico approaches including docking, molecular dynamic simulations and the MM(GB/PB)SA method for the investigation of ligand binding have been described extensively in literature to further supplement the understanding of the underlying mechanism of ligand recognition by the CYP isoforms [36,87,88,89,90,91,92]. The MM(GB/PB)SA method is commonly used for the calculation of binding free energies based on the MD simulation trajectories [76]. The MM(GB/PB)SA analysis and per residue energy decompositions for a set of CYP3A4 substrates used clinically in combinatorial cancer therapy (cytarabine, daunorubicin, doxorubicin and vincristine) has shown the importance of Asp61, Asp76, Ala174, Glu122, Asp214, Arg105, Arg212, Ala217, Glu234, Ala305, Glu308, Ala370, Ile369 and Glu374 residues for favorable interactions with ligands [36]. Herein, the WaterSwap results were analyzed further to find the contribution of 13 important binding site residues to the binding of the inhibitors. Arg106, Arg212, Phe213, Glu308, Ala370, Arg372, Gly481, Leu483 and Gln484 play a major role in the stabilization of the selected CYP3A4-inhibitor complexes. Interactions with Arg105, Ser119, Thr309 and Glu374 are unfavorable as they show a preference for the water cluster rather than the inhibitor. This information could be useful to help guide the development of these lead molecules to enhance binding. Principally, the WaterSwap method is fully automated and robust, providing new tools for the analysis and visualization of the important driving forces in binding of protein–ligand complexes and is broadly applicable to study binding or assess the impact of mutations on binding [76,77,82,83].

## 3. Materials and Methods

### 3.1. Ligand Selection

For the identification of highly potent inhibitors of CYP3A4, a large dataset of CYP3A4 inhibitors was extracted from the ChEMBL database (https://www.ebi.ac.uk/chembl/) [93] using a filtering criteria of IC_50_ ≤ 100 µM. After removal of duplicates and fragments the remaining data set of 2747 compounds was subjected to LipE- and LE calculations [22] as described by Hopkins et al. [49]. A brief detail of LipE and LE calculations of the CYP3A4 inhibitors has been provided in the Supplementary Materials (SM) (Section S1, Figure S1a,b and Table S1). Finally, five CYP3A4 inhibitors showing optimal LipE and LE values were selected for further MD studies to estimate the binding free energy and stability of the inhibitor-bound complexes.

### 3.2. Molecular Docking Studies 

CYP3A4 inhibitors were built in GaussView 5 [94] and their geometry optimized using density functional theory with the B3LYP functional and the 6–31G(d) and 6–31 + G(2d,p) basis sets. The 2.05 Å resolution crystal structure of human CYP3A4 1TQN [44] was retrieved from the protein data bank to provide the starting point for docking and MD simulations. The missing 1TQN H-I loop residues were built as described by Webb and Sali [95] using Modeller version 9.15 [48]. The modeled structure of CYP3A4 (1TQN) [44] was prepared using AutoDockTools [63] where polar hydrogens were added after computing the Kollman partial charges and solvent parameters for the macromolecule. After protein modification, non-polar and polar hydrogens were merged, Gasteiger charges were computed for optimized ligands and the flexible torsions were defined.

The CYP3A4 binding site is large and highly promiscuous allowing multiple ligands to bind simultaneously [96]. Additionally, for CYP3A4 two different binding cavity volumes (1,386 Å^3^ and 520 Å^3^) have been reported previously [44,71]. Therefore, in order to provide the appropriate docking area and dimensions of the CYP3A4 binding site, the Grid Box pointer was centered above the heme at grid points x: −20, y: −21 and, z: −11 with a grid size of 60 × 60 × 60 Å and a grid spacing of 0.375 Å. The conformational space of ligands was explored using a Lamarckian genetic algorithm (LGA) that is based on a local search method for the energy minimization [97]. AutoDock evaluates conformations during docking simulations using a semi-empirical free energy force field that accounts for the intramolecular and desolvation terms along with directionality in hydrogen bonds [98]. The free energy scoring function involves two steps; firstly, the evaluation of the intramolecular energetics for the transition from the unbound state to the bound conformation for each molecule separately (Equation (1)), followed by the intermolecular energetics evaluation of bringing the two molecules together into the bound complex (Equation (2)) [99].

(1) $$\Delta G=\left(V_{Bound}^{L-L}-V_{Unbound}^{L-L}\right)+\left(V_{Bound}^{P-P}-V_{Unbound}^{P-P}\right)+\left(V_{Bound}^{P-L}\right)-V_{Unbound}^{P-L}+\Delta S_{conf}$$

Here, *V* indicates the six pairwise evaluations, *ΔS_conf_* gives an estimate of the conformational entropy lost upon binding, *L* and *P* refer to the ligand and protein in a protein–ligand complex. Equation (2) includes a pairwise atomic term in the evaluation of dispersion/repulsion, hydrogen bonding, electrostatics, and desolvation:

(2) $$\begin{array}{c} V=W_{vdw}\sum_{i,j}(\frac{A_{ij}}{r_{ij}^{12}}-\frac{B_{ij}}{r_{ij}^{6}})+W_{hbond}\sum_{i,j}E\left(t\right)(\frac{C_{ij}}{r_{ij}^{12}}-\frac{D_{ij}}{r_{ij}^{10}})+\;W_{elec}\sum_{i,j}\frac{q_{i}q_{j}}{\varepsilon \left(r_{ij}\right)r_{ij}}\\ +W_{sol}\sum_{i,j}(S_{i}V_{j}+S_{i}V_{j})e^{\frac{-r_{ij}^{2}}{2\sigma^{2}}} \end{array}$$

A maximum of 10 different conformations were generated for each ligand during the docking run and the minimum energy pose was selected for further investigation. The CYP3A4-inhibitor interaction profiles were analyzed to assess whether the binding conformations agree with available data reported for other CYP3A4 inhibitors. The stability of the selected CYP3A4-inhibitor docked complexes was evaluated using MD simulations.

### 3.3. Molecular Dynamic Simulations using Graphical Processing Units (GPUs)

The unliganded 1TQN structure and the finally selected inhibitor-bound complexes were prepared using the LEaP module in AMBER14 [65]. All protein complexes were solvated using TIP3PBOX [99] with a margin of 12.0 Å and an appropriate number of Cl^−^ ions were added to neutralize the overall charge on the system. The GAFF force field [100] was used to parameterize the ligands using the ANTECHAMBER program with AM1-BCC charge assignment method [65], while the proteins were modeled using AMBER force field FF14SB [101]. The topology and coordinate files for the entire system were generated by the LEaP module of AMBER 14 [65]. Heme parameters were adopted from an earlier study [102] and the bond between CYS442 SG atom and Fe atom of heme was explicitly defined.

MD simulations of the selected conformers were performed after optimizing the positions of hydrogen atoms, water molecules and all atoms within each complex by energy minimizations through the SANDER module in AMBER14 [65]. MD simulations were performed using a 2 fs time step with a cutoff radius of 8.0 Å for nonbonded interactions. The particle-mesh Ewald method [103] was used for the calculation of long-range electrostatic interactions and all bonds containing hydrogen were constrained using the SHAKE algorithm [104]. Heating was performed by gradually increasing the temperature from 0 to 300 K over a period of 20 ps. Langevin temperature control with a random number assignment to each trajectory (random initial velocities) was used. A 100 ps equilibration was carried out at a temperature of 300 K with 1 atm pressure which was followed by 140 ps under the same temperature and pressure conditions. A 50 ns MD simulation was performed for all the complexes using PMEMD.CUDA from AMBER14 [65]. Trajectory analysis was performed using the CPPTRAJ module in AMBER14 [65]. The stability of each CYP3A4-inhibitor complex was assessed by evaluating the C_α_RMSD, C_α_RMSF, Rg and hydrogen bond analysis with respect to the starting structures.

### 3.4. Binding Energy Predictions Using WaterSwap

The WaterSwap method from the Sire package [40] was used for the prediction of absolute binding free energies for the CYP3A4-inhibitor docked complexes. The conformational landscapes of the CYP3A4-inhibitor complexes obtained after the molecular dynamics simulations were clustered using CPPTRAJ [65] based on the hierarchical agglomerative approach using average-linkage [105]. Clustering was performed based on the RMSD of important active site residues including Tyr53, Phe57, Asp76, Arg105, Arg106, Phe108, Ser119, Arg212, Phe215, Ile301, Phe304, Ala305, Thr309, Ile369, Ala370, Met371, Arg372, Glu374, Leu482 and Leu483 with no fit. For each complex the centroid of these clusters was used as the starting structure for WaterSwap calculations [40].

The WaterSwap method calculates the absolute protein–ligand binding free energy using an explicit water model by essentially swapping the ligand dimensions with an equal volume of water within the binding site [40]. The method takes into account the protein–water, ligand–water and protein–water–ligand interactions thus, removing the double decoupling cavitation problems existing in the implicit solvent methods for the estimation of binding free energies [76]. The WaterSwap method uses the Monte Carlo simulations [106] for attaining the WaterSwap reaction coordinates (WSRC) representative of the ligand swapping with water and also for the calculation of binding free energies [40]. The WSRC is augmented with the identity constraint [107] that identifies a cluster of water molecules equivalent to the volume and shape of the ligand within the protein binding site which is achieved by coupling two periodic boxes including the protein box (protein–ligand complex solvated in water box) and the bulk-water-box (box of water centered on the ligand coordinates) to the same thermostat and barostat [40], followed by the subsequent swapping of the two systems using a dual topological approach [108,109]. Equation (5) is used for the calculation of binding energy of all systems:

(3) $$\begin{array}{c} E\left(\lambda \right)=E_{proteinbox}+E_{waterbox}+E_{ligand}+E_{cluster}+\left(1-\lambda \right)(E_{ligand:proteinbox}\\ +E_{cluster:waterbox})+\left(\lambda \right)\left(E_{cluster:proteinbox}+E_{ligand:waterbox}\right) \end{array}$$

where, *E_proteinbox_* is the energy of all molecules within the protein excluding the ligand, *E_waterbox_* is the energy of all molecules in the water box excluding the water cluster, *E_ligand_* denotes the intramolecular energy of the ligand, *E_cluster_* represents the interaction energy between all the water molecules present within the water cluster, *E_ligand:proteinbox_* shows the interaction energy between all atoms of the protein box and the ligand, and *E_cluster:waterbox_* is the interaction energy between all water molecules in bulk water box and the identified water cluster.

The reaction involves simultaneous decoupling of the ligand from the protein box and the water cluster from the water box followed by parallel coupling of the ligand to the water box and the water cluster to the protein box. *λ* is the WaterSwap reaction coordinate that transforms from *λ* = 0 to *λ* = 1 to assist the transfer of the ligand from the protein bound state (at *λ* = 0) to the water box (at *λ* = 1). Additionally, the transformation also corresponds to the transfer of water cluster from water box to fill the resultant cavity in the protein box. For a WaterSwap calculation of a system, the absolute binding free energy is estimated using replica exchange thermodynamic integration method (RETI) [77,110,111] that takes into account replica exchange moves along the WSRC on the basis of calculated energy gradients using finite-difference TI (FDTI) with respect to *λ* (Equation (5)). However, to calculate the free energy gradients along *λ*, the collective averages are computed using Equation (5).

(4) $$dE/d\lambda =(E_{cluster:proteinbox}+E_{ligand:waterbox})-(E_{ligand:waterbox}+E_{cluster:waterbox})$$

(5) $$dG/d\lambda_{\lambda }=dE/d\lambda_{\lambda }$$

Monte Carlo simulations [110] are used to accept or reject neighboring replicas periodically during the replica exchange, thus allowing full sampling of ligand-bound and ligand-free states during the simulation of the connected protein box/bulk-water-box. Moreover, the quality of the WaterSwap estimations is also dependent on the number of Monte Carlo simulations used to calculate the energy gradients across *λ*. The potential of mean force (PMF) along the WSRC was obtained by the integration of the energy gradients along *λ* using Equation (7) [40].

(6) $$G_{Bind}=\;-\frac{1}{0}DG/d\lambda_{\;\lambda \;}d\;\lambda$$

The ligand unbinding from the protein is represented as the negative integral in WSRC. The absolute binding free energy in WaterSwap is based on a single reaction coordinate which is approximated by averaging the total energy gradient along *λ* to achieve free energy gradient followed by the integration over the entire WSRC. Since the WaterSwap method for calculating binding free energy is based on averages of the gradients of total WaterSwap energies across the sampled configurations, it is therefore also reasonable to average the gradients of the total energy components (*G_proteinbox_*, *G_waterbox_*) with respect to *λ*. These decompositions are approximations and as a result, *G_proteinbox_* and *G_waterbox_* will not be exactly equal to *G_bind_*. Therefore, the decomposition of components would assist in revealing whether a favorable binding free energy is due to ligand showing a strong affinity for protein or a low affinity for water [76].

Finally, in this study the most representative cluster for each complex was selected for further analysis, where the ligands were swapped with the representative water clusters after the construction of WaterSwap coordinates for the calculation of the PMF using the RETI method. For all CYP3A4-inhibitor-bound complexes WaterSwap calculations were performed for 1000 iterations using 25 million moves of Monte Carlo (MC) sampling across each of the 16 *λ*s (0.005, 0.071, 0.203, 0.269, 0.335, 0.401, 0.467, 0.533, 0.599, 0.665, 0.731, 0.797, 0.863, 0.929, 0.995) at a temperature of 298.15 K and pressure of 1 atm on each replica. For the WaterSwap calculations the MC across the 16 *λ* windows have been used since it is already reported that sixteen MC simulations are adequate, generating sixteen free energy gradients spaced across *λ* [76]. The free energy of binding was evaluated using three different statistical techniques: free energy perturbation (FEP), thermodynamic integration (TI) and the Bennett’s acceptance ratio (BAR) algorithm. Furthermore, the degree of convergence of the calculated results can be assessed by considering the level of agreement between these three energy values, where ideally the deviation should be within 1 kcal/mol [76].

Furthermore, the application of MM/PBSA and MM/GBSA methods to assure the accurate ranking of inhibitors on the basis of their binding energy can add value to the drug design research. The binding energies of the five CYP3A4 inhibitor docked complexes were estimated through (MM/PBSA) and (MM/GBSA) method using Equations (7) and (8) [41,42].

(7) $$\Delta G_{binding}=G_{complex}-G_{protein}-G_{ligand}=\Delta E_{MM}+\;\Delta G_{PB}+\Delta G_{nonpolar}-T\Delta S$$

(8) $$\Delta \Delta G_{binding}=\Delta E_{MM}-\Delta G_{sol}+\Delta G_{SA}$$

Herein, the *ΔE_MM_* accounts for the difference between the minimized energies of the CYP3A4 inhibitor-bound complexes and the total energies of the enzyme and inhibitor (including electrostatic and the van der Waals energies), *TΔS* shows the change in entropy of the ligand binding conformations, *G_solv_* is the summation of contributions from the polar states and *ΔG_SA_* parameter accounts for the difference in the surface area energies and is estimated from the solvent accessible surface area (SASA) (using a water probe radius of 1.4 Å).

## 4. Conclusions

The CYP3A4 binding site is highly promiscuous in nature and therefore, it is more prone to drug–drug interactions due to the inhibition or induction of the metabolic enzyme. The interaction of CYP3A4 with a broad spectrum of chemical entities leads to greater chances of inhibition. Thus, highly potent inhibitors were selected to elucidate the mechanism of CYP3A4 inhibition and extend the understanding of CYP3A4 mediated drug–drug interactions. The current study provides an overview of the most probable binding poses and energy values of CYP3A4 inhibitors which may further help to address the primary question of CYP3A4 inhibition. Ultimately, the probable binding modes and interaction profiles of the selected CYP3A4 inhibitors were explored by docking and MD simulations. Binding site residues including Phe57, Arg105, Arg106, Ser119, Arg212, Phe213, Thr309, Ser312, Ala370, Arg372, Glu374, Gly481 and Leu483 were observed to be significant in terms of interactions with CYP3A4. The binding energies were calculated within considerable range using MM/GBSA, MM/PBSA and the WaterSwap methods. The WaterSwap calculations demonstrate that the highly potent inhibitors show an overall greater affinity towards CYP3A4 and the binding site residues Arg106 and Arg372 favor the stabilization of the all CYP3A4-inhibitor complexes. The ultimate goal of this study and a follow up study is to design a pipeline for CYP3A4 inhibition to be used in the lead optimization programs. However, herein, we present ligand binding and the estimation of free energy of binding with CYP3A4. The estimation of free energy could pave the way towards understanding the strength of CYP inhibition. In a follow up study the ligand–protein complexes with minimum binding free energies could be used as a reference/template for building predictive models for CYP3A4 inhibition that may have advantage over the already existing predictive models due to the use of more appropriate templates based on binding free energy. This study provides an understanding of CYP3A4 inhibition process and our results could guide the use of multiple approaches, including simulations, relevant to CYP3A4 inhibition for the virtual screening of new chemical entities during lead optimization programs, to provide basis for early screening of CYP inhibitors ultimately leading to the design/selection of new chemical entities with suitable ADME-Tox properties and reduced side effects.

## Figures and Tables

**Table 1:** Ligand–protein interaction profiles of the selected CYP3A4 inhibitors within the CYP3A4 binding site.

CYP3A4 Inhibitors	Autodock Energy (kcal/mol)	van der Waals Interactions Before MD	Hydrogen Bond Interactions in the Docked Poses			van der Waals Interactions in Centroid Structure from Clustering	Hydrogen Bond Interactions in the Centroid Structures			van der Waals Interactions after MD	Hydrogen Bond Interactions at 50 ns		
			Acceptor Atom	Donor Atom	Distance Å		Acceptor Atom	Donor Atom	Distance Å		Acceptor Atom	Donor Atom	Distance Å
CYP3A4-YK1	−9.9	Leu482, Thr309, Phe304, Asp76, Thr224, Arg372, Leu373, Arg375	Lig: Sulphonyl=O3 Lig: Carbonyl=O10	Arg212-NH1 Arg105-NE	3.01 2.93	Phe220, Gly109, Pro107, Leu373, Tyr53, Thr224, Glu374, Gly481, Ala370, Leu482, Arg212	Arg372=O Lig: Carbonyl=O3 Lig: Carbonyl=O3	Lig: Hydroxyl-O2 Arg105-NE Glu374-NH	2.62 3.68 3.70	Tyr53, Arg105, Arg106, Pro107, Gly109, Phe213, Leu216, Phe220, Ala370, Leu373, Gly481, Leu482	Arg372=O Lig: Carbonyl=O3	Lig: Hydroxyl-O2 Glu374-NH	2.58 3.05
CYP3A4-YK2	−11.6	Phe316, Glu308, Gln484, Thr309, Phe304, Phe108, Arg105, Phe213, Glu374, Gly481, Leu482, Leu483	Ser312-OG Ala370=O Arg372=O	Lig: Hydroxyl-O Lig: Hydroxyl-O2 Lig: Hydroxyl-O2	3.01 2.81 3.13	Ser312, Glu308, Arg212, Thr309, Phe304, Phe108, Arg105, Phe57, Glu374, Leu373, Leu483, Gln484	Arg372=O	Lig: Hydroxyl-O2	2.71	Phe57, Arg105, Arg212, Phe304, Thr309, Ser312, Leu373, Glu374, Leu462, Gln484	Arg372=O	Lig: Hydroxyl-O2	2.96
CYP3A4-YK3	−9.5	Phe57, Arg105, Arg106, Phe108, Ala305, Phe304, Thr309, Met371	Lig: Sulphonyl=O2 Lig: Carbonyl=O1 Lig: Carbonyl=O1 Lig: Carbonyl=O1	Arg212-NH1 - Ser119-OG Arg212-NH1 Arg212-NH2	3.05 3.53 3.18 3.02	Ile50, Tyr53, Asp76, Leu216, Leu221, Thr224, Val225, Gly481, Leu482, Arg212, Ala370, Leu373	Lig: Sulphonyl=O1 Lig: Hydroxyl-O3 Lig: N2	Arg106:NE Arg105:NH1 Arg372:NH	3.11 3.20 3.50	Asp76, Ile47, Arg105, Phe215, Leu216, Phe220, Leu221, Thr224, Ala370, Leu373, Glu374, Leu482	No hydrogen bonds formed	No hydrogen bonds formed	--
CYP3A4-YK4	−11.7	Phe108, Thr309, Phe304, Glu308, Ser312, Phe316, Leu373, Met371, Leu482,	Ser312-OG Leu483=O	Lig: Amine-N5 Lig: Amine-N5	3.16 3.12	Ser312, Gln484, Leu482, Thr309, Asp214, Phe316, Leu483, Pro485, Pro368, Met371, Ala370, Phe108, Ser119, Arg105	Phe213=O Arg212-NE Arg212-NH1	Lig: Amine-N2 Lig: Amine-N3 Lig: Amine-N3	3.22 3.64 3.66	Phe57, Arg106, Phe215, Phe241, Ile301, Pro368, Ala370, Glu374, Gly480, Gly481, Leu482	Ser119-O	Lig: Hydroxyl-O1	2.96
CYP3A4-YK5	−10.4	Arg105, Arg106, Ser119, Phe241, Ile301, Phe215,	Glu374-OE2 Lig: Carbonyl=O3	Lig: Hydroxyl-O Arg212-NH1	3.32 3.13	Phe316, Ile369, Leu483, Met371, Arg372, Phe215, Glu374, Ser312, Gln484, Glu308,	Lig: Carbonyl=O Ala349=O Lig: Carbonyl=O1	Leu483-NH Lig: Amine-N2 Arg212:NH1	2.82 3.38 2.91	Arg105, Ser119, Phe304, Gly306, Glu308, Ser312, Phe316, Ile369, Ala370, Met371, Gln484, Pro485	Thr309-OG1 Lig: Carbonyl=O	Lig: Hydroxyl-O3 Leu483-N	2.77 3.00

The terms MD and Lig are used as an abbreviation for Molecular Dynamics and Ligand respectively in Table 1.

**Table 2:** Binding free energies of the selected inhibitor-bound complexes (CYP3A4-YK1 toCYP3A4-YK5) from Monte Carlo calculations in WaterSwap, MM/PBSA and MM/GBSA. Note that two different WaterSwap calculations (using centroid clusters c0 and c1) were performed for YK4 and YK5 as the dominant cluster did not include structures from the entire simulation.

Inhibitor-Bound Complex					WaterSwap			
	IC_50_ nM	Autodock Score kcal/mol	MM/PBSA kcal/mol	MM/GBSA kcal/mol	BAR kcal/mol	FEP kcal/mol	TI kcal/mol	Average kcal/mol
CYP3A4-YK1	0.1	−9.9	−40.78 ± 0.43	−61.22 ± 0.43	−41.1	−40.2	−40.6	−40.6 ± 0.5
CYP3A4-YK2	0.4	−11.6	−25.50 ± 0.33	−37.44 ± 0.24	−37.5	−36.8	−37.7	−37.3 ± 0.5
CYP3A4-YK3	10	−9.5	−32.37 ± 0.31	−49.48 ± 0.31	−47.3	−46.3	−46.5	−46.7 ± 0.5
CYP3A4-YK4	2.6	−11.7	−30.87 ± 0.31	−34.96 ± 0.23	−40.3	−39.5	−39.3	−39.7 ± 0.5
					−31.6	−30.6	−30.5	−30.9 ± 0.6
CYP3A4-YK5	38	−10.4	−22.52 ± 0.34	−38.46 ± 0.25	−36.1	−36.3	−35.6	−36.0 ± 0.4
					−40.4	−39.8	−39.9	−40.0 ± 0.3

## Supplementary Materials

Supplementary materials can be found at https://www.mdpi.com/1422-0067/20/18/4468/s1.
