# Supplementary material for: Molecular Dynamics Simulation Framework to Probe the Binding Hypothesis of CYP3A4 Inhibitors
Source: Int J Mol Sci. 2019 Sep 10;20(18):4468. doi: 10.3390/ijms20184468 (PMC6769491; doi:10.3390/ijms20184468)
Supplement: Supplementary file 1 [file ijms-20-04468-s001.pdf]

SUPPORTING INFORMATION

Molecular Dynamics Simulation framework to probe the binding hypothesis of CYP3A4 inhibitors

Yusra Sajid Kiani[1], Kara E. Ranaghan[2], Ishrat Jabeen[*1] and Adrian J. Mulholland[*2]

1. Research Center for Modeling and Simulation (RCMS), National University of Science and Technology (NUST), Sector H-12, 44000, Islamabad, Pakistan
2. Centre for Computational Chemistry, School of Chemistry, University of Bristol, Bristol BS8 1TS, U.K

* Correspondence: Corresponding Author: Ishrat.jabeen@rcms.nust.edu.pk, adrian.mulholland@bristol.ac.uk

Contents

Section S1: Ligand selection......3-4

Figure S1......5

Figure S2......14

Section S2: RMSD analysis of MD replicates......7

Figure S3......8

Figure S4......9

Figure S5......10

Figure S6......10

Figure S7......11

Figure S8......12

Figure S9......13

Figure S10......14

Figure S11......15

Table S2......16

References 17

Section S1: Ligand selection

A large dataset of CYP3A4 inhibitors was extracted from the ChEMBL database and the Lipophilic efficiency (LipE) and Ligand efficiency (LE) calculations were performed for the selection of highly potent inhibitors of CYP3A4 subtype. The ligand efficiency metrics provide an estimation of the lipophilicity, size and overall molecular properties that determine the binding affinity of a drug towards its particular target [1]. The LipE parameter provides the normalization of the potency (pIC_{50}) for a compound's lipophilicity (clogP/logD) against a particular target and is calculated by subtracting clogP/logD from the pIC_{50} (equation1). For LipE profiling the clogP values were calculated using fragment based method implemented in Bio-Loom software package [2].

$$\mathbf{LipE = LLE = pIC_{50}(pKi/pKd) - clogP} \qquad \textbf{(eq 1)}$$

The Ligand efficiency metric delivers a balance of potency and molecular size in terms of a ligand binding to its target and is measured as the ratio of binding free energy (ΔG) to the number of heavy atoms (HA) [3]. Free energies (ΔG) for CYP3A4 inhibitors were calculated using equation 2 where, R is the gas constant and T is the absolute temperature. ΔG calculations were performed by substituting IC_{50} values for K_d, as anticipated by Hopkins *et al* which is also approved by the experimental studies of Kuntz and co-workers [3, 4].

$$\mathbf{\Delta G = -RT\ lnK_d} \qquad \textbf{(eq 2)}$$

A temperature of 310K was used to compute Ligand efficiencies of the CYP3A4 inhibitors in kcal/mol/heavy atom using equation 3.

$$\mathbf{LE = (\Delta g) = -\ {}^{\Delta G}/_{HA(non\text{-}hydrogen\ atom)}} \qquad \textbf{(eq 3)}$$

Reynolds fitting procedure [5] was adopted to achieve a size independent fit quality score (LigE_Scale) for the CYP3A4 inhibitor dataset. This was accomplished using a modified exponential function to fit top Ligand efficiencies against the number of heavy atoms (eq 4). The Fit Quality (FQ) scoring function was formulated by finding the ratio of LE of ligands to the normalized LE_Scale (eq 5).

$$\mathbf{LE\_Scale = 1.5 * EXP(-0.053 * HA)} \quad \textbf{eq 4}$$

$$\mathbf{FQ = LE/LE\_Scale} \qquad \textbf{eq 5}$$

Reynolds *et al* [5] put forward that high FQ score near 1 or above, is indicative of optimal binding however, lower FQ scores show suboptimal binding. Overall, a LipE ≥5, clogP values of ~1-3, IC_{50} ~10-150 nM, LE ≥0.29 (kcal/mol/heavy atom) and FQ ≥1 were used as a selection criteria to identify highly efficient inhibitors of CYP3A4. Finally, five CYP3A4 inhibitors fulfilling the selection criteria were further used for docking and molecular dynamic simulations.

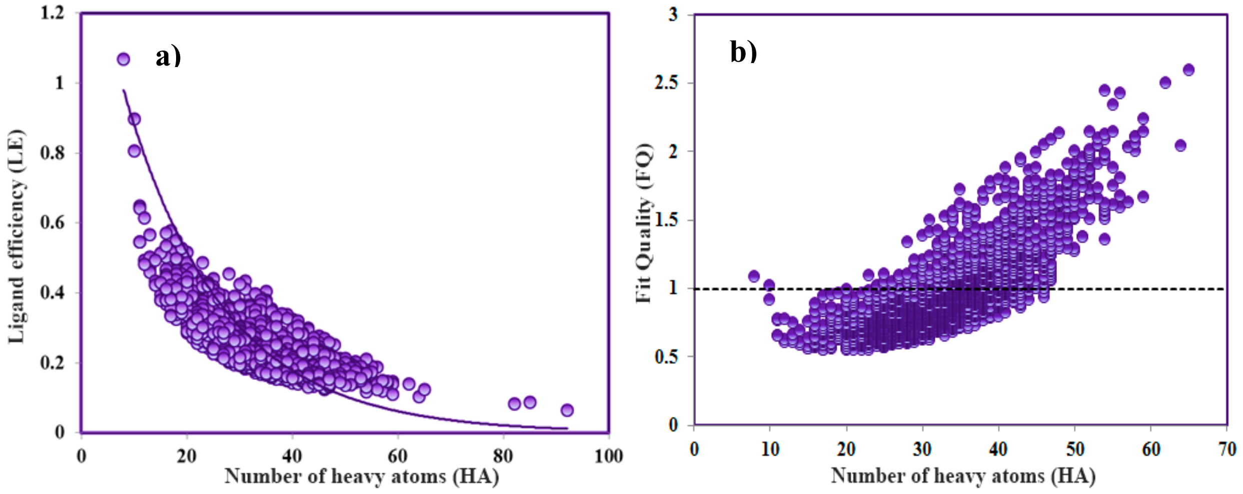

Figure S1. a) Ligand efficiency profiling and **b)** Fit quality plots of CYP3A4 inhibitors.

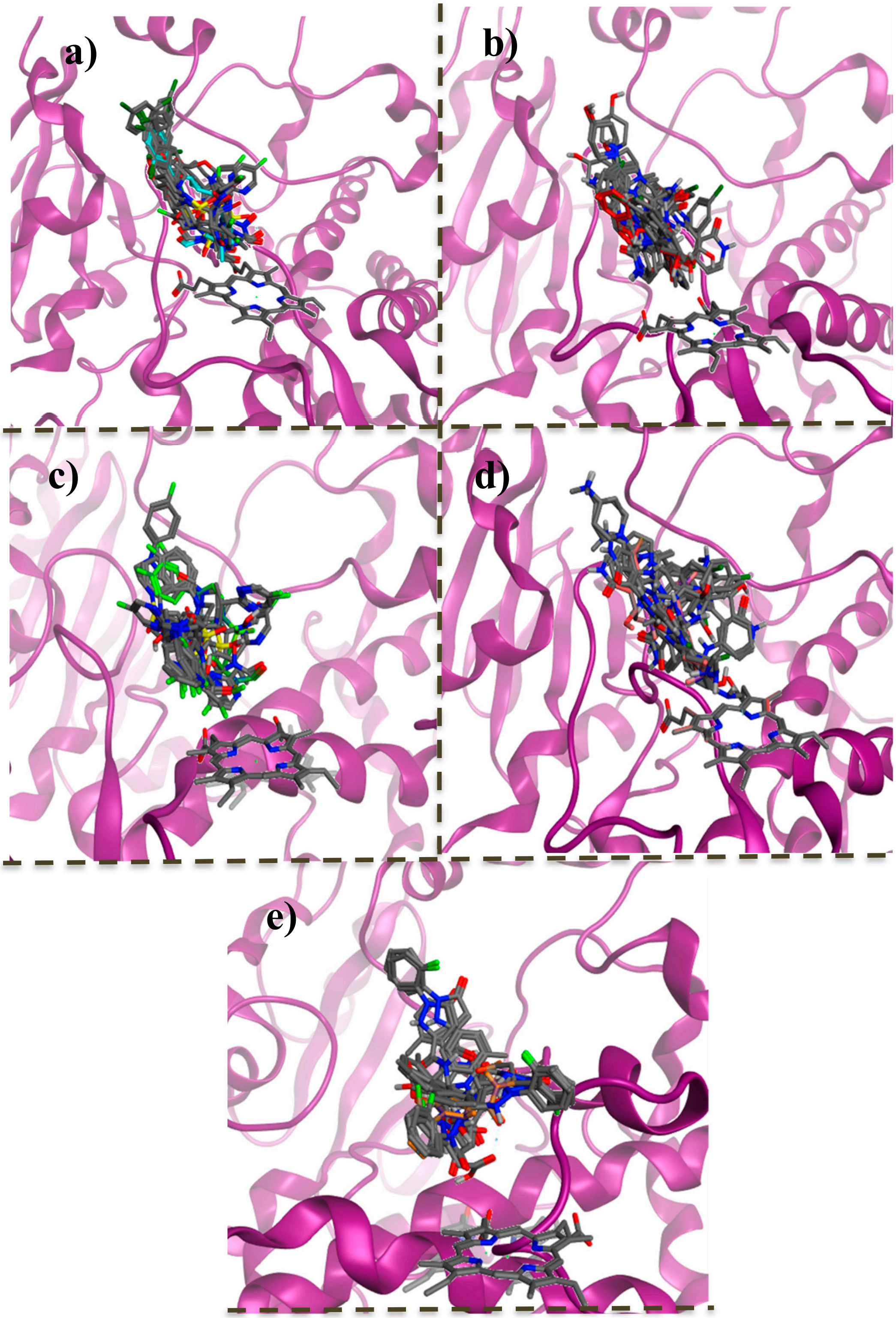

Figure S2. The docked clusters obtained using AutoDock for a) CYP3A4-YK1, b) CYP3A4-YK2, c) CYP3A4-YK3, d) CYP3A4-YK4 and e) CYP3A4-YK5. Here the selected minimum energy poses for CYP3A4-YK1-YK5 are shown in cyan, red, green, blue and orange respectively.

Section S2: RMSD analysis of MD replicates of the selected CYP3A4-inhibitor complexes

The conformational analysis of the replicated MDs for the ligand bound complexes (CYP3A4-YK1-YK5) was performed. The average C_{α}RMSD values for the replicated MDs of CYP3A4-YK1-YK5 complexes differ by ~0.09 to 0.57 Å as shown by the average C_{α}RMSD values of 3.13(± 0.7) Å (CYP3A4-YK1-MD1): 3.33(± 0.4) Å (CYP3A4-YK1-MD2), 2.23(± 0.4) Å (CYP3A4-YK2-MD1): 2.80(± 0.6) Å (CYP3A4-YK2-MD2), 2.11(±0.6) Å (CYP3A4-YK3-MD1): 2.65(± 0.5) Å (CYP3A4-YK 3-MD2):, 2.09(± 0.3) Å (CYP3A4-YK4-MD1): 2.18(±0.4) Å (CYP3A4-YK4-MD2) and 2.31(± 0.7) Å (CYP3A4-YK5-MD1): 2.50(± 0.7) Å (CYP3A4-YK5-MD2). The C_{α}RMSD plots for the two MD runs of the selected CYP3A4 inhibitor bound complexes are shown in Figure S3 Therefore, the initial run of MDs (MD1) with smaller average C_{α}RMSD and Rg values were considered final for further analysis.

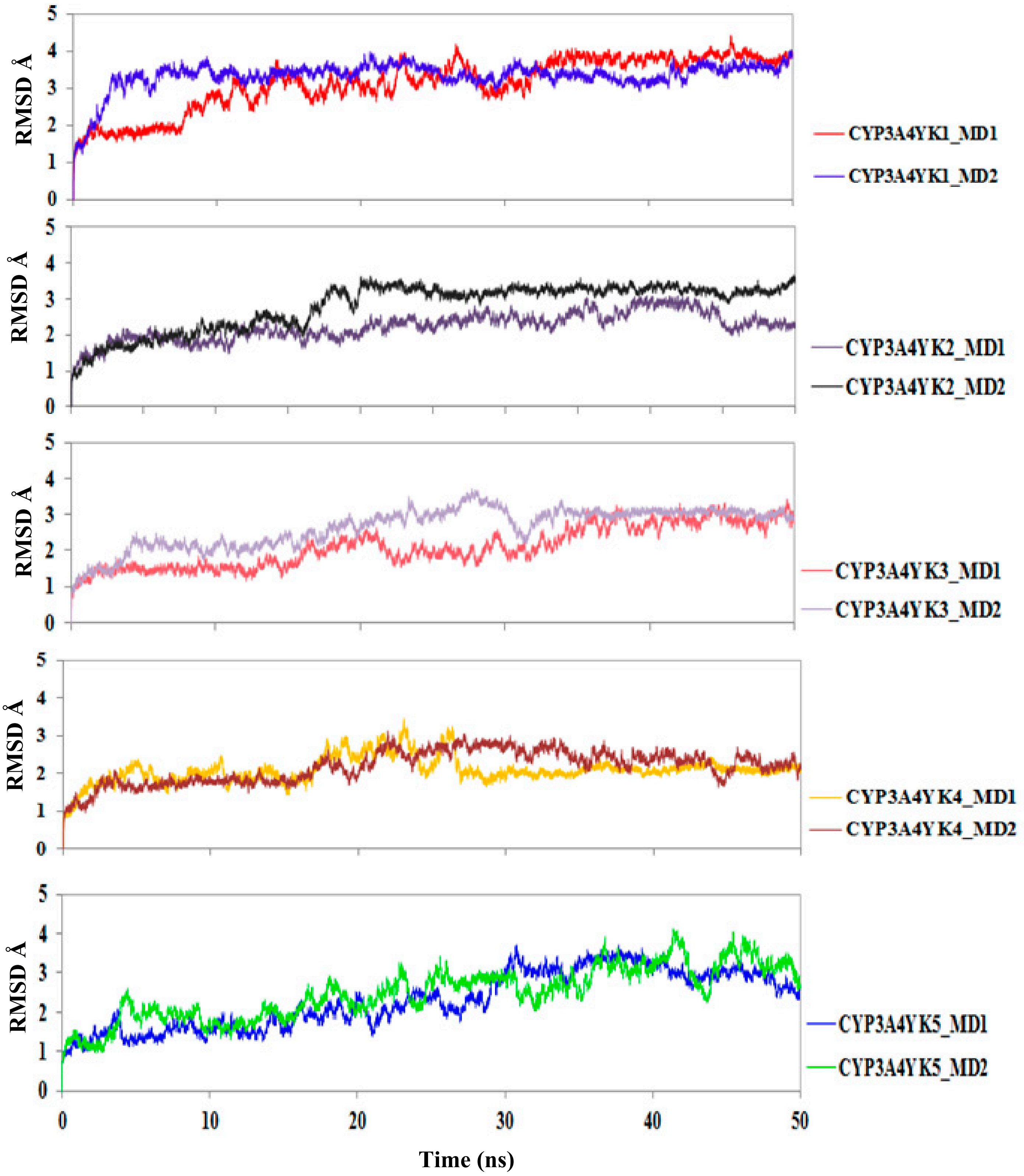

Figure S3. The CαRMSD of the two MD runs of the five selected CYP3A4-inhibitor complexes (CYP3A4-YK1-YK5) during the 50 ns MD simulations.

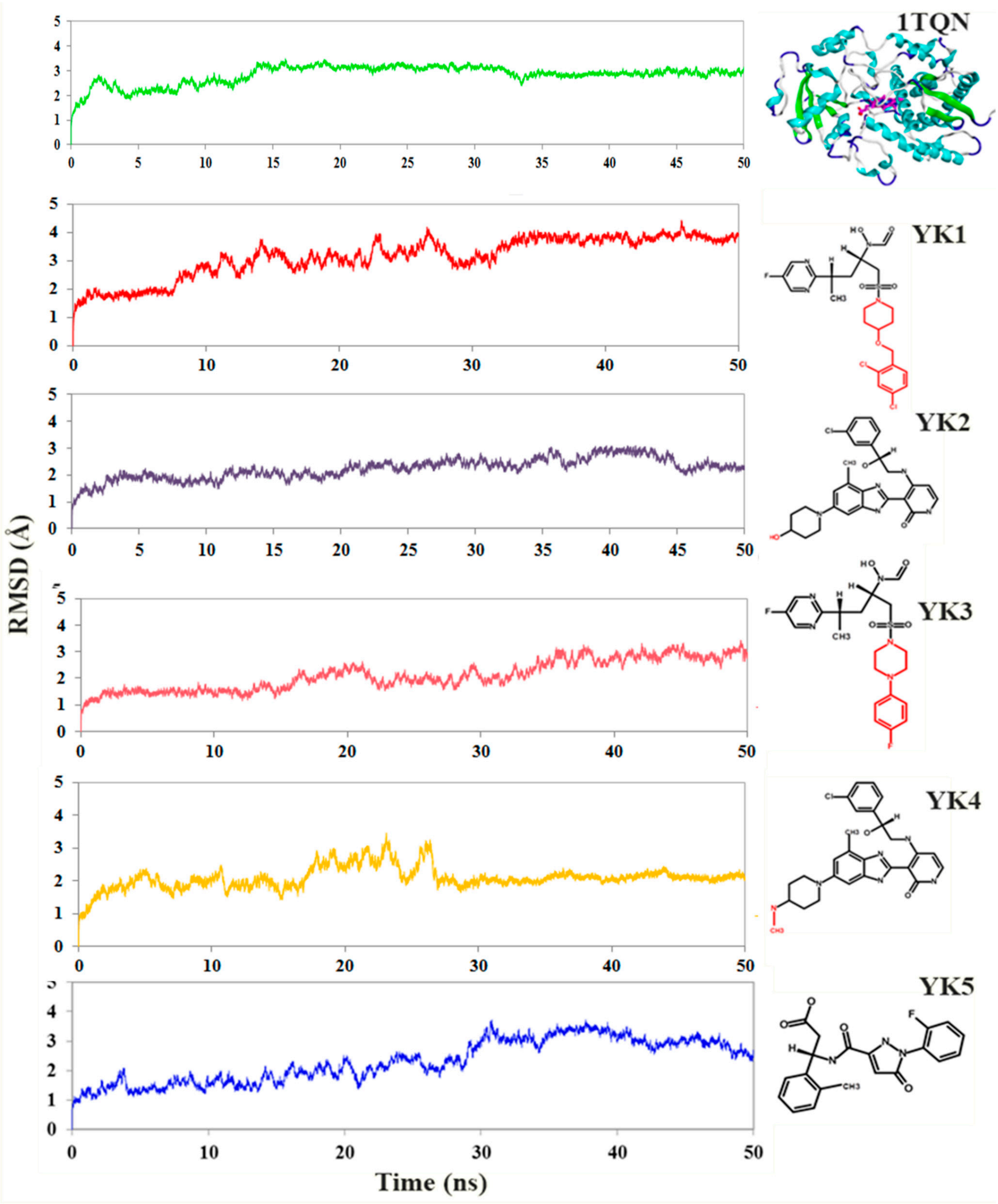

Figure S4. The CαRMSD of the apo-state CYP3A4 (1TQN) and the five selected CYP3A4-inhibitor complexes (CYP3A4-YK1-YK5) during the 50 ns MD simulations.

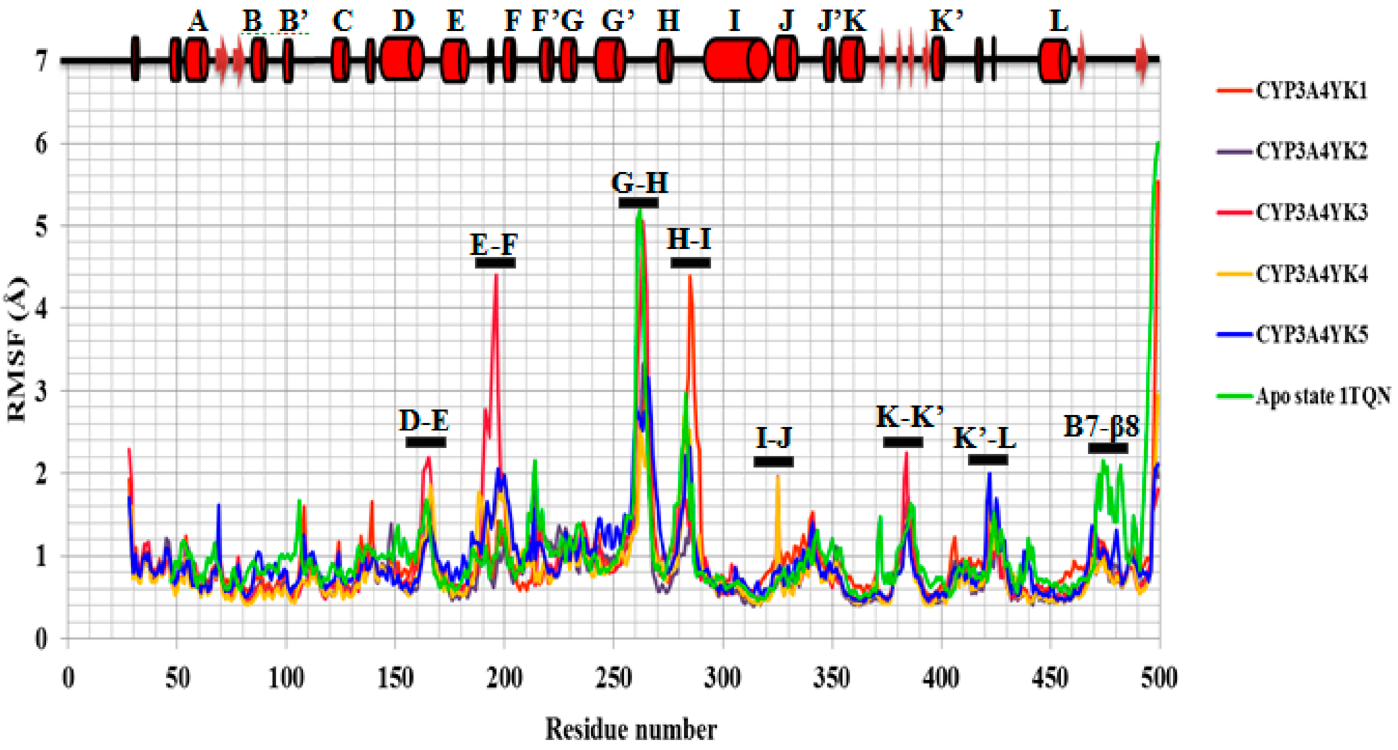

Figure S5. The Cα RMSF of the individual residues of the apo-state CYP3A4 (1TQN) and the five selected CYP3A4-inhibitor complexes (CYP3A4-YK1-YK5) during the 50 ns MD simulations

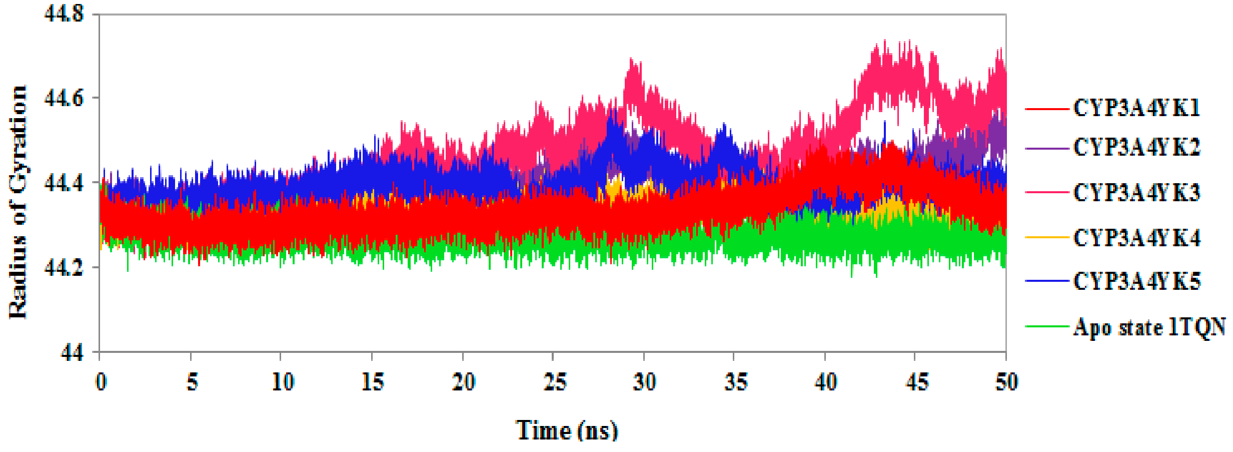

Figure S6: The radius of gyration (Rg) for the apo-state CYP3A4 (1TQN) and the five selected CYP3A4-inhibitor complexes (CYP3A4-YK1-YK5) during the 50 ns MD simulations.

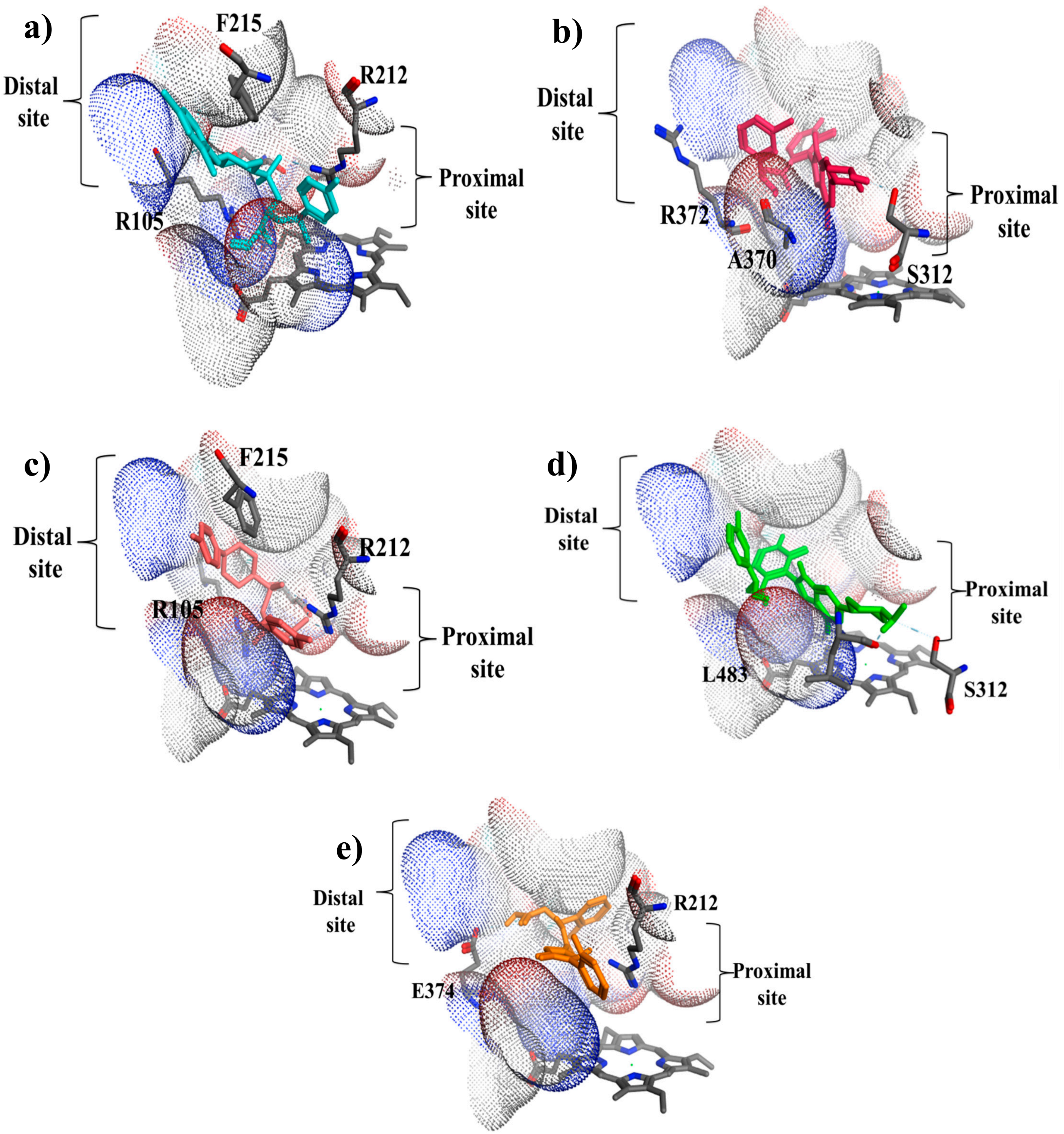

Figure S7. The docked conformations of the selected minimum energy poses of **a)** CYP3A4-YK1, **b)** CYP3A4-YK2, **c)** CYP3A4-YK3, **d)** CYP3A4-YK4 and **e)** CYP3A4-YK5 complexes with the distal and proximal binding sites shown as discussed by Tanaka et al.

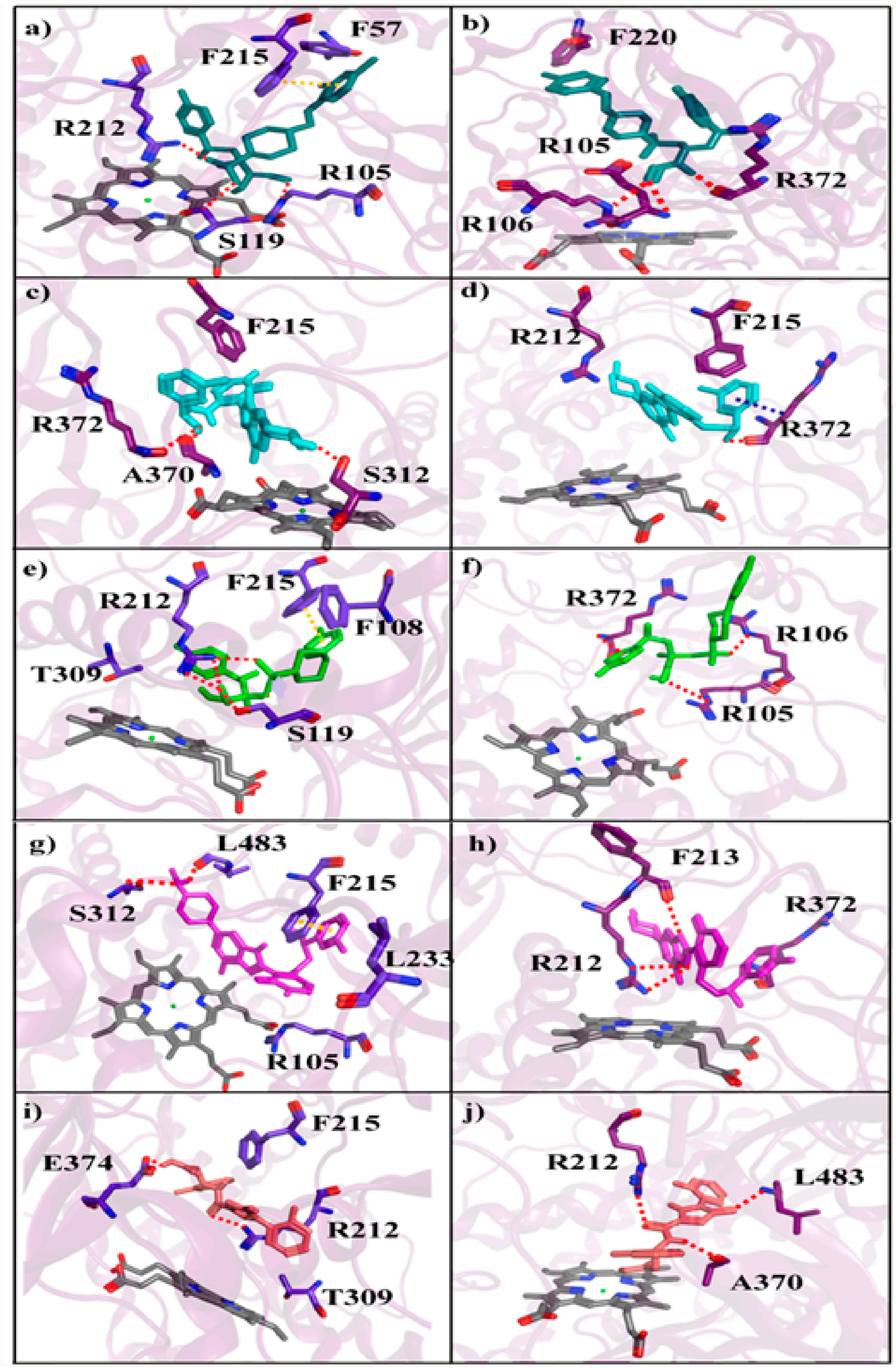

Figure S8. The two columns show the interaction pattern of the selected inhibitor complexes **a)** CYP3A4-YK1 docked pose, **b)** CYP3A4-YK1 centroid structure **c)** CYP3A4-YK2 docked pose, **d)** CYP3A4-YK2 centroid structure, **e)** CYP3A4-YK3 docked pose, **f)** CYP3A4-YK3 centroid structure, **g)** CYP3A4-YK4 docked pose, **h)** CYP3A4-YK4 centroid structure, **i)** CYP3A4-YK5 docked pose and **j)** CYP3A4-YK5 centroid structure. The dotted lines show hydrogen bonds (red), pi-pi stacking (yellow) pi-cation (green) and pi-H (blue)

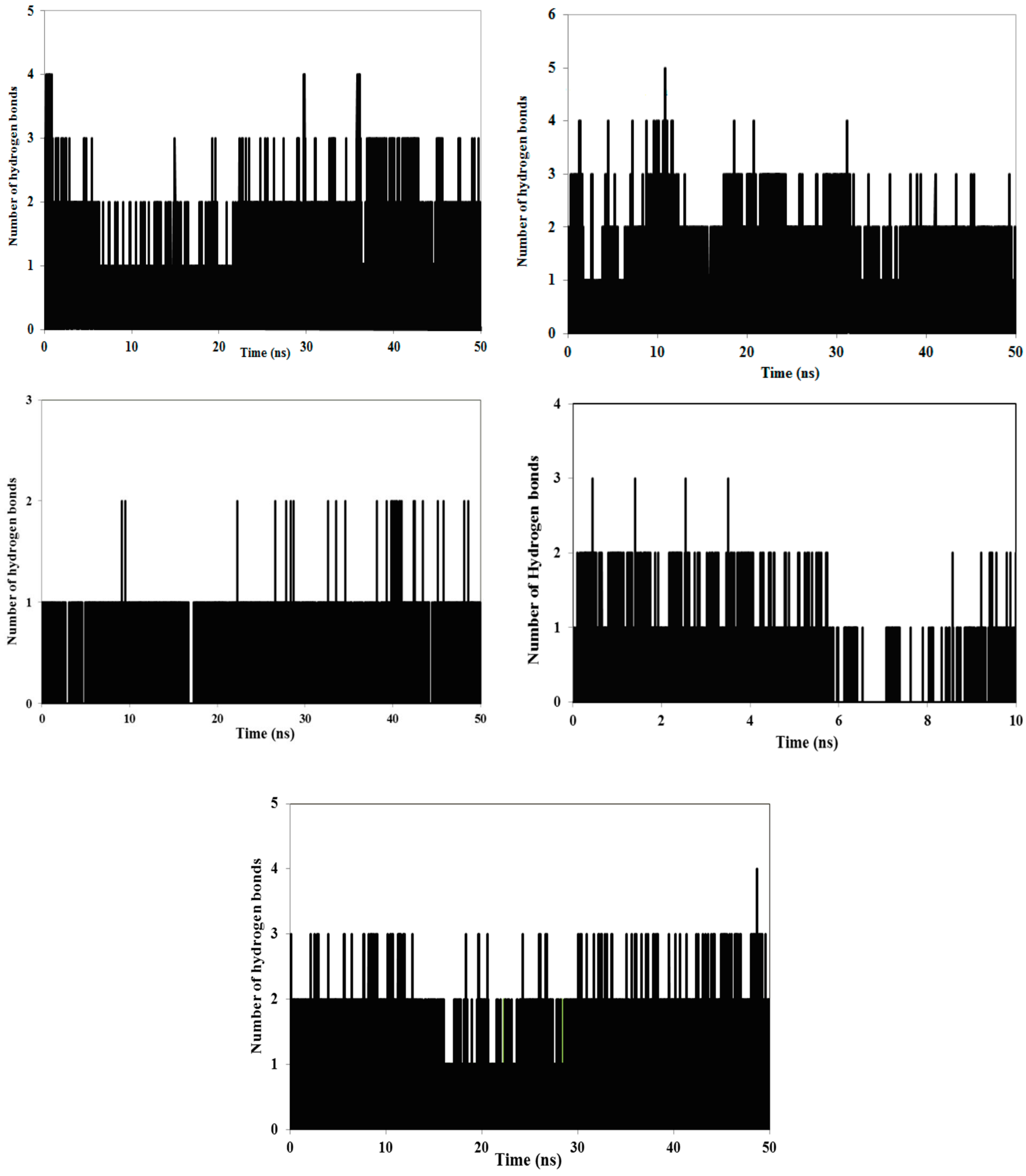

Figure S9. The time dependent analysis of the number of hydrogen bonds formed between CYP3A4 and the selected inhibitors in **a)** CYP3A4-YK1, **b)** CYP3A4-YK2, **c)** CYP3A4-YK3, **d)** CYP3A4-YK4 and **e)** CYP3A4-YK5 complexes.

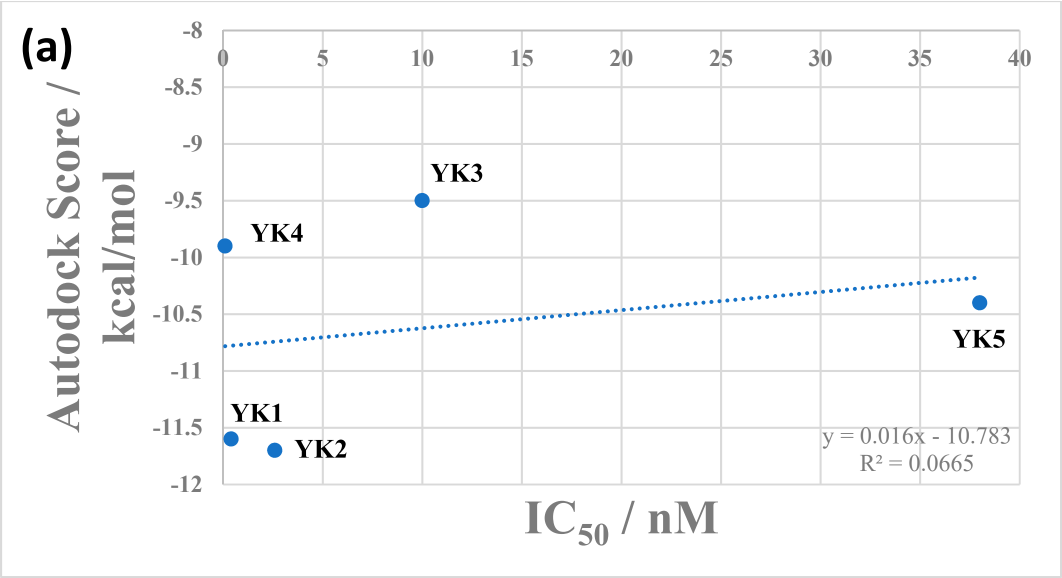

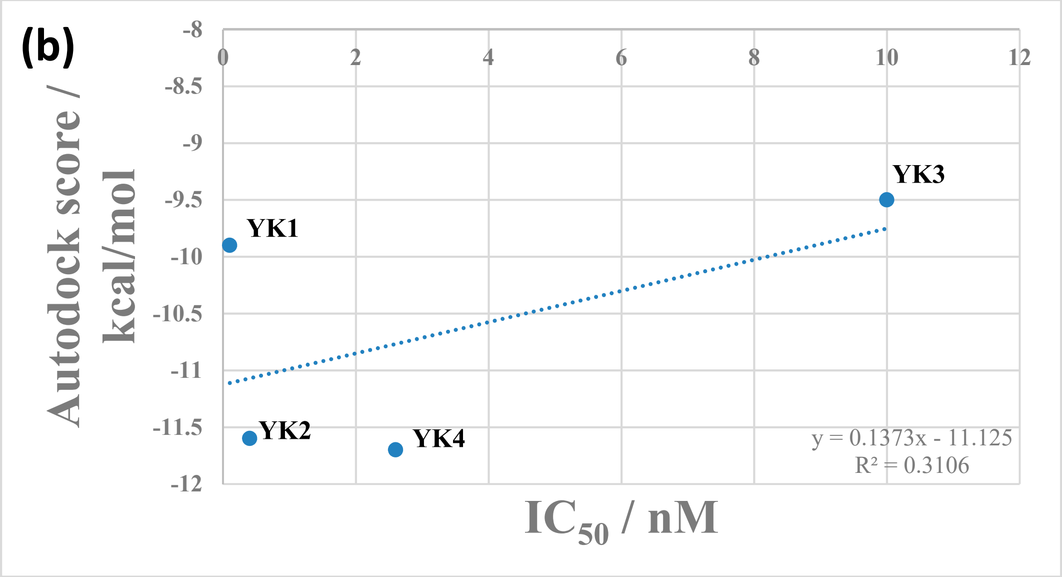

Figure S10. a) The correlation between the docking score for the complex predicted by Autodock and the IC_{50} values for YK1-5, b) The correlation between the docking score for the complex predicted by Autodock and the IC_{50} values with the data for YK5 removed as it is a significant outlier compared to the other compounds

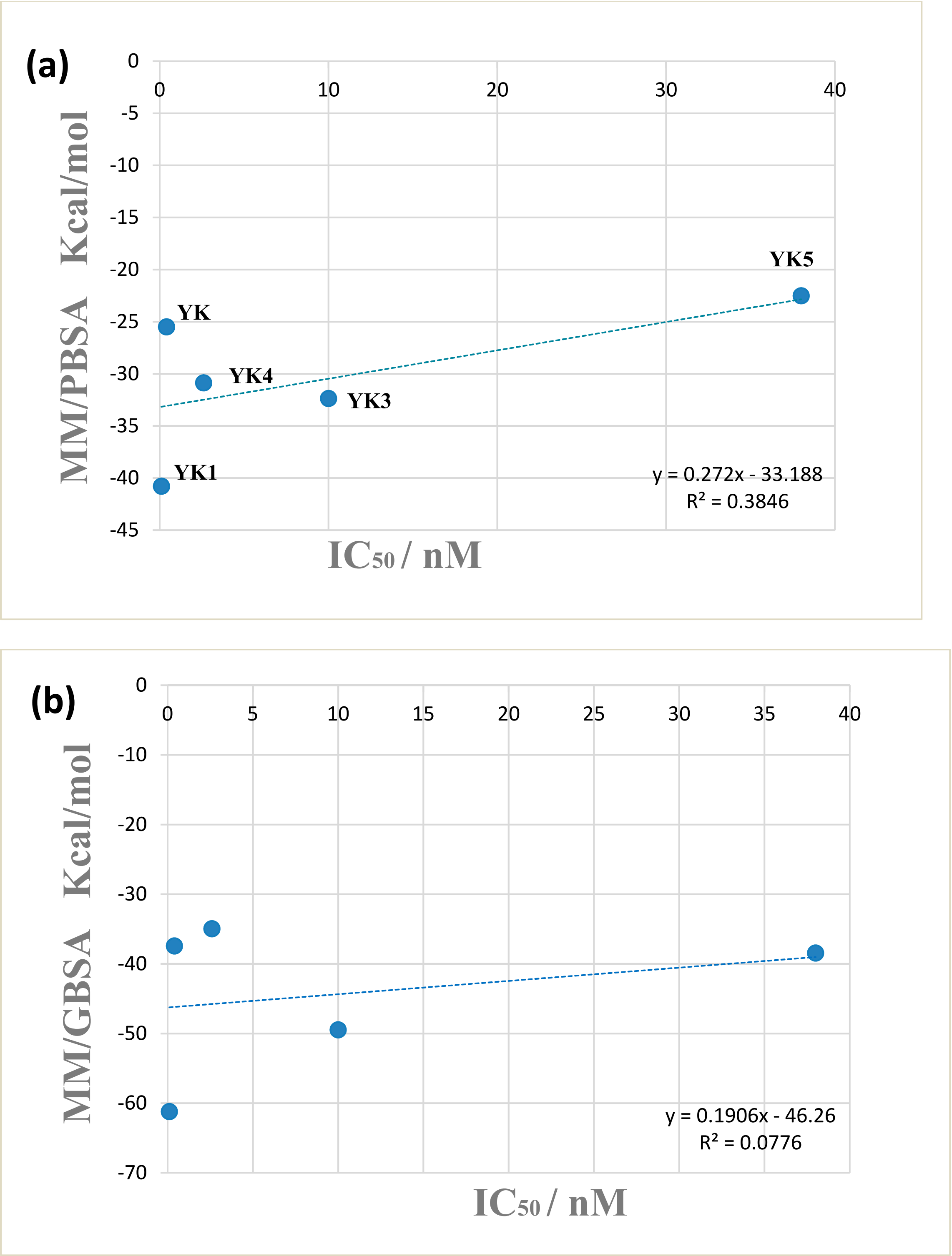

Figure S11. a) The correlation between the binding free energy of the complexes predicted by MM/PBSA and the IC_{50} values for YK1-5, b) The correlation between the binding free energy of the complex predicted by MM/GBSA and the IC_{50} values for YK1-5.

Table S2: MM/PBSA and MM/GBSA based binding energy profiles (with energy components) of CYP3A4-inhibitor bound complexes.

Energy Components	CYP3A4-YK1		CYP3A4-YK2		CYP3A4-YK3		CYP3A4-YK4		CYP3A4-YK5	
	MM/GBSA	MM/PBSA	MM/GBSA	MM/PBSA	MM/GBA	MM/PBSA	MM/GBS A	MM/PBSA	MM/GBSA	MM/PBSA
ΔE_{vdw}	–69.30±0.26	–-69.30±0.26	–57.92±0.24	–57.92±0.24	– 59.57±0.23	–59.57±0.23	–53.45±0.21	–53.45±0.21	–52.31±0.24	–52.31±0.24
$\Delta E_{electrostatic}$	–43.66±0.59	–43.66±±0.59	–18.13±0.56	–18.13±0.56	–41.72±0.45	–41.72±0.45	–6.09±0.25	–6.09±0.25	–13.97±0.35	–13.97±0.35
$\Delta E_{EGB/EPB}$	59.84±0.31	77.30±0.37	45.59±0.40	55.86±0.38	59.53±0.34	73.86±0.37	31.58±0.22	34.76±0.39	33.49±0.28	48.47±0.34
$\Delta E_{nonpolar}$	–8.11 ±0.02	–5.13±0.008	–6.98±0.01	–5.31±0.008	–7.72±0.016	–4.94±0.007	–6.50±0.02	–6.09±0.01	–5.67±0.01	–4.71±0.008
ΔG_{gas}	– 112.96±0.63	–112.96±0.63	–76.05±0.49	–76.05±0.49	–101.29±0.045	–101.29±0.45	–59.54±0.35	–59.54±0.35	–66.28±0.30	–66.28±0.30
ΔG_{solv}	51.73 ±0.31	72.17±0.37	38.61±0.40	50.56±0.38	51.81±0.34	51.84±0.38	25.08±0.21	28.67±0.34	27.82±0.28	43.77±0.33
ΔG_{total}	–61.22 ±0.43	–40.79±0.43	–37.44±0.24	–25.49±0.33	–49.40±0.31	–32.37±0.31	–34.46±0.23	–30.87±0.38	–38.46±0.25	-22.52±0.34

References

1. Hopkins, A.L., et al., *The role of ligand efficiency metrics in drug discovery.* Nature Reviews Drug Discovery, 2014. **13**(2): p. 105-121.
2. Jones, P.M. and A.M. George, *Mechanism of ABC transporters: a molecular dynamics simulation of a well characterized nucleotide-binding subunit.* Proceedings of the National Academy of Sciences, 2002. **99**(20): p. 12639-12644.
3. Hopkins, A.L., C.R. Groom, and A. Alex, *Ligand efficiency: a useful metric for lead selection.* Drug discovery today, 2004. **9**(10): p. 430-431.
4. Kuntz, I., et al., *The maximal affinity of ligands.* Proceedings of the National Academy of Sciences, 1999. **96**(18): p. 9997-10002.
5. Reynolds, C.H., S.D. Bembenek, and B.A. Tounge, *The role of molecular size in ligand efficiency.* Bioorganic & medicinal chemistry letters, 2007. **17**(15): p. 4258-4261.
